# Effects of Instrumentality and Personal Force on Deontological and Utilitarian Inclinations in Harm-Related Moral Dilemmas

**DOI:** 10.3389/fpsyg.2020.01222

**Published:** 2020-06-19

**Authors:** Jonas Ludwig, Rainer Reisenzein, Anette Hiemisch

**Affiliations:** ^1^Institut für Psychologie, Universität Greifswald, Greifswald, Germany; ^2^Fachbereich Staats- und Gesellschaftswissenschaften, Zeppelin Universität Friedrichshafen, Friedrichshafen, Germany; ^3^Pädagogische Psychologie, Pädagogische Hochschule Weingarten, Weingarten, Germany

**Keywords:** moral dilemmas, moral judgment, process dissociation procedure, personal force, instrumentality of harm

## Abstract

Moral dilemmas often concern actions that involve causing harm to others in the attempt to prevent greater harm. But not all actions of this kind are equal in terms of their moral evaluation. In particular, a harm-causing preventive action is typically regarded as less acceptable if the harm is a means to achieve the goal of preventing greater harm than if it is a foreseen but unintended side-effect of the action. Likewise, a harm-causing preventive action is typically deemed less acceptable if it directly produces the harm than if it merely initiates a process that brings about the harmful consequence by its own dynamics. We report three experiments that investigated to which degree these two variables, the instrumentality of the harm (harm as means vs. side-effect; Experiments 1, 2, and 3) and personal force (personal vs. impersonal dilemmas; Experiments 2 and 3) influence deontological (harm-rejection) and utilitarian (outcome-maximization) inclinations that have been hypothesized to underly moral judgments in harm-related moral dilemmas. To measure these moral inclinations, the process dissociation procedure was used. The results suggest that the instrumentality of the harm and personal force affect both inclinations, but in opposite ways. Personal dilemmas and dilemmas characterized by harm as a means evoked higher deontological tendencies and lower utilitarian tendencies, than impersonal dilemmas and dilemmas where the harm was a side-effect. These distinct influences of the two dilemma conceptualization variables went undetected if the conventional measure of moral inclinations, the proportion of harm-accepting judgments, was analyzed. Furthermore, although deontological and utilitarian inclinations were found to be largely independent overall, there was some evidence that their correlation depended on the experimental conditions.

## Introduction

Moral dilemmas often concern actions that involve causing harm to others in the attempt to prevent greater harm. To illustrate, consider the following scenario taken from Foot (1967; slightly modified): You are a doctor almost out of stock of the drug required to treat a patient with severe symptoms. There is only one dose left that would suffice to cure this person; but the same dose could also be used to cure five other patients who have the same deadly disease at a much earlier stage. Would you split up the dose to help the five less seriously ill patients, knowing that the other patient will die without the medication? And would you judge it as morally appropriate if someone else in your position would do so?

Philosophers, psychologists, and neuroscientists have made extensive use of thought experiments of this kind to understand how people evaluate others’ behavior and choose own actions in moral dilemmas. This research has identified several features of moral dilemmas that influence these evaluations and decisions (see, e.g., Christensen et al., 2014). Two of the most important of these dilemma conceptualization variables (Christensen and Gomila, 2012) are the instrumentality of the harm and personal force (e.g., Cushman et al., 2006; Greene et al., 2009; Feltz and May, 2017).

The *instrumentality of the harm* concerns the question whether the harm caused is a means to, or a side-effect of the agent’s attempt to prevent greater harm. The above example illustrates the second case: The death of the seriously ill patient is a foreseen but unintended side-effect of splitting the last dose of the drug between five other patients. The majority of respondents accept harmful actions of this kind, but reject them if harm is a means (Foot, 1967; O’Neill and Petrinovich, 1998; Hauser et al., 2007; Waldmann and Dieterich, 2007; Greene et al., 2009; see Feltz and May, 2017, for a review). For example, if the only way to save the lives of five patients were to transplant five different organs taken from another patient, most people will object to that action, even though it involves the same trade-off.

*Personal force* refers to the role of the agent’s activity in causing harm to another person in the attempt to prevent greater harm. This factor has given rise to a distinction between personal and impersonal moral dilemmas. In personal moral dilemmas, the agent initiates and executes the harm-causing action at first hand. Again, the above example may illustrate this case: The doctor personally withholds the medication from the first patient to administer it to the other patients. In impersonal moral dilemmas, in contrast, the agent sets off a process which brings about the harmful consequences by itself (e.g., mediated by a mechanical apparatus), without any further activity of the agent beyond the initial impetus. To illustrate, imagine that the drugs are administered to patients by a computer-controlled intravenous delivery system, and that the doctor only needs to enter a command to cause this system to redirect the medication away from the first patient to the other five patients. It has been found that harmful actions performed to prevent greater harm are typically regarded as unacceptable in personal dilemmas, but as acceptable in impersonal dilemmas (see, e.g., Greene et al., 2001; Greene et al., 2004; Cushman et al., 2006; Moore et al., 2008; Greene et al., 2009; Moore et al., 2011).

Although the effects of instrumentality and personal force on moral judgments and decisions are well-established, the intervening mental processes responsible for these effects are as yet not fully understood. In particular, it is as yet not known how, precisely, the effects of instrumentality and personal force relate to two conflicting motivational tendencies that are generally thought to be evoked by moral dilemmas of the described type: The tendency to avoid causing harm to others, and the tendency to maximize the overall benefit for oneself and others. As noted by several authors (e.g., Greene, 2007; Waldmann and Dieterich, 2007), these two action tendencies are in line with what is declared to be the correct behavior in dilemma situations by, respectively, the deontological, and utilitarian schools of moral philosophy (see also Greene, 2007, 2014; Conway et al., 2018a). According to deontologists, the moral appropriateness of an action depends solely on the type of action it is, independent of the circumstances and hence, regardless of its consequences in a specific situation (e.g., killing another person is always morally inappropriate). In contrast, according to utilitarianists, the morally appropriate action is always the action that, in a given situation, maximizes the benefit to others and oneself. Conway and Gawronski (2013) therefore call the two described action tendencies *deontological* and *utilitarian inclinations*, and judgments that agree with these inclinations, deontological and utiliarian judgments. We follow this terminology in the present article, but note that this is not meant to imply that these inclinations are based on a person’s explicit commitment to a deontological vs. utilitarian moral philosophy; this may, but need not be the case (see Conway et al., 2018a).

If one accepts that evaluative judgments and decisions in moral dilemmas are proximately caused by deontological and utilitarian inclinations, then instrumentality and personal force should influence these judgments and decisions by affecting one or both of these inclinations. What is presently still unclear is the exact nature of this influence: Are the effects of instrumentality and personal force on moral evaluations and choices mediated by changes in harm-rejection tendencies, benefit-maximization tendencies, or both? The main goal of the present research is to clarify this question.

To derive predictions about the effects of instrumentality and personal force on deontological and utilitarian inclinations in moral dilemmas, we consulted theories about the processes that underlie these motivational tendencies. One of the most prominent theories is the dual-process model of moral judgment first proposed by Greene et al. (2001), and further elaborated by Greene (2007, 2014; Greene et al., 2009). This theory assumes that judgments and choices in moral dilemmas depend on two processes: A largely automatic and fast affective process, and a more deliberative, slower reasoning process. More precisely, Greene et al. (2001) proposed that contemplating certain kinds of harmful actions (in particular, harmful actions in personal dilemmas) automatically triggers negative affect, which evokes an action tendency to reject the harmful action. This tendency then causes deontological moral judgments and actions.

But moral dilemmas can also trigger a reasoning process, in which the person considers the possible consequences of the different action alternatives available to her, evaluates them, and integrates the evaluations into an overall judgment or decision. This deliberative process is assumed to typically lead to utilitarian judgments and actions (Greene, 2007, 2014; see also Conway et al., 2018a).

Greene’s (2007) theory has inspired numerous empirical studies (e.g., Greene et al., 2001, 2004, 2008, 2009; Mendez et al., 2005; Cushman et al., 2006; Ciaramelli et al., 2007; Hauser et al., 2007; Koenigs et al., 2007; Waldmann and Dieterich, 2007; Moore et al., 2008; Conway and Gawronski, 2013; Conway et al., 2018a; see Greene, 2014, for a review). These studies have provided considerable evidence in support of the dual-process theory. Nevertheless, recent research suggests that the processes underlying judgment and decision making in moral dilemma situations may be more complex than assumed in Greene’s (2007) original model (see also Crockett, 2013; Cushman, 2013). In particular, there is now evidence which suggests that affective processes influence not only deontological, but also utilitarian judgments (Reynolds and Conway, 2018; see also Cushman et al., 2010; Baron et al., 2018), and likewise, that deliberative processes influence not just utilitarian but also deontological judgments (Gamez-Djokic and Molden, 2016; Byrd and Conway, 2019).

In light of these findings, less rigid versions of the dual-process model have been proposed (e.g., Skitka and Conway, 2019). These “softer” versions of the dual-process model abandon the assumption of the original theory that there are exclusive links between affect and deontological inclinations, and deliberation and utilitarian inclinations, as well as the assumption, also made in the original theory, that the affective process always occurs first. However, the newer versions of the dual-process theory still uphold the assumption that deontological inclinations are primarily based on aversive feelings evoked by contemplating harmful actions, whereas utilitarian inclinations are primarily based on deliberations about outcome maximization (see also Cushman, 2013; Conway et al., 2018a). This “softened” version of the dual-process model served as the theoretical basis of the present studies.

What do the dual-process model and the associated research predict about the effects of instrumentality and personal force on deontological and utilitarian inclinations? Findings of Greene et al. (2001, 2004, 2009) suggest that high instrumentality (harm as a means) and high personal force (i.e., personal dilemmas) both cause negative affect about the contemplated action, whereas low instrumentality (harm as a side-effect) and low personal force (i.e., impersonal dilemmas) do not evoke strong emotions. In addition, findings by Greene et al. 2001, 2004 suggest that impersonal dilemmas are more likely to evoke deliberative processes than personal dilemmas. Based on these findings, we predicted that the instrumentality of the harm influences deontological but not utilitarian inclinations, whereas personal force influences deontological and utilitarian inclinations in opposite ways.

To test these hypotheses, which are further elaborated below, a method is needed that allows the separate quantification of deontological and utilitarian inclinations in moral dilemmas. The conventionally used measure of moral inclinations, the percentage of utilitarian responses (i.e., responses that approve of the harmful action), is not suited for this purpose, as it reflects the joint influence of both tendencies on a single dimension. Therefore, we resorted to a different measurement approach first used to study moral dilemmas by Conway and Gawronski (2013): the process dissociation procedure (PD).

## The Process Dissociation Approach to Moral Judgment

The process dissociation procedure (PD) was originally developed in memory research (Jacoby, 1991), but has proven to be useful for examining the interplay of different cognitive processes in many other research areas (see Payne and Bishara, 2009), including several areas of judgment and decision making (e.g., Payne, 2001; Ferreira et al., 2006; Conway and Gawronski, 2013). The goal of the PD procedure is to provide quantitative estimates of the relative contributions of two (or more) hypothesized mental processes to a behavior or judgment of interest. The basic idea behind PD is to learn about these cognitive processes by comparing situations in which the outcomes of the processes are aligned (i.e., favor the same response) to situations in which they are in conflict (favor different responses). To achieve this goal, two (or more) different versions of the experimental materials must be designed that conform with the procedure’s requirement of comparing congruent conditions (aligned processes) to incongruent conditions (conflicting processes).

Conway and Gawronski (2013) were the first to apply the PD procedure to moral judgments in dilemma situations. As in the studies reported in the present article, their aim was to disentangle the contributions of deontological and utilitarian inclinations to moral judgments, and to study how experimental manipulations known to affect moral judgments affect the two kinds of inclinations. Following the logic of the PD procedure, Conway and Gawronski (2013) presented participants with a set of moral dilemmas in which deontological and utilitarian inclinations either favored the same action (congruent scenarios) or different actions (incongruent scenarios). For example, the *incongruent version* of the “car accident” dilemma used by Conway and Gawronski (2013, p. 231) was as follows:

You are driving through a busy city street when all of a sudden a young mother carrying a child trips and falls into the path of your vehicle. You are going too fast to break in time; your only hope is to swerve out of the way. Unfortunately, the only place you can swerve is currently occupied by a little old lady. If you swerve to avoid the young mother and baby, you will seriously injure or kill the old lady. Is it appropriate to swerve and hit the old lady in order to avoid the young mother and child?

In this situation, the tendency to reject causing harm regardless of outcomes (deontological inclination) suggests to morally condemn swerving, even though not swerving will cause even greater harm (the serious injury or death of the mother and her child). In contrast, the tendency to maximize the overall benefit (utilitarian inclination) suggests to approve of swerving, as this action, while harming the old lady, will save the mother and her child.

**FIGURE 1:**
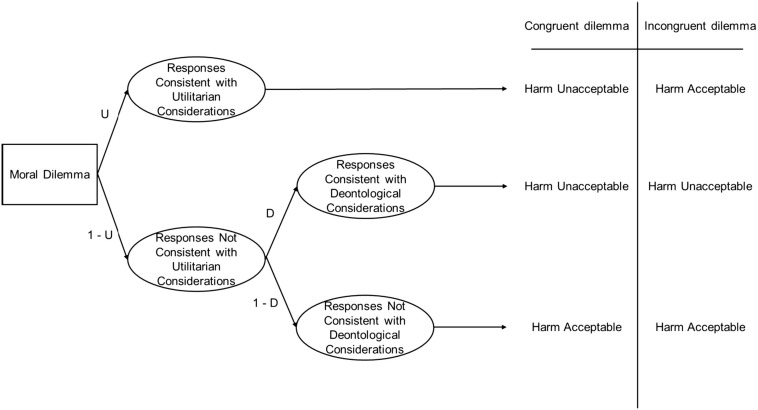
Processing tree for congruent and incongruent trials. U = PD Utilitarianism parameter, D = PD Deontology parameter. For congruent dilemmas, this process model implies that *p*(harm unacceptable) = U + (1–U) × D, and for incongruent dilemmas, that *p*(harm unacceptable) = (1–U) × D. These equations can be solved for U and D, yielding U = *p*(harm unacceptable | congruent) – *p*(harm unacceptable | incongruent), and D = *p*(harm unacceptable | incongruent) / (1-U). Hence, the parameters can be algebraically determined. Figure reprinted from Conway et al. (2018a), with permission from Elsevier.

In contrast, in the *congruent version* of the car accident scenario, the participants were informed that the only available place for swerving was occupied by a group of children. Under these circumstances, swerving to avoid injuring the young mother and her child is not only rejected by the person’s deontological inclination (the harm-rejection tendency), but also by her utilitarian inclination (outcome-maximization tendency), because not swerving now also produces the greatest overall benefit. Figure 1 shows the hypothesized cognitive processes and resulting judgments for congruent and incongruent moral dilemmas.

The participants of Conway and Gawronski’s (2013) experiments were presented with ten incongruent and ten matched congruent moral dilemmas. The responses given to these scenarios allowed to estimate, for each participant, the probability of accepting vs. not accepting the harmful action in the congruent and incongruent dilemmas. Based on these data and the processing tree shown in Figure 1, two parameters were estimated for each person, one representing the person’s deontological inclination (D) and the other her utilitarian inclination (U; see the caption to Figure 1 and Conway et al., 2018a, for the details of the parameter estimation procedure). These parameters were used as dependent variables in subsequent analyses, designed to find out how they were influenced by experimental manipulations and how D and U correlated with each other and with covariates of interest.

Conway and Gawronski (2013) found that experimental manipulations of cognitive load and empathic concern had selective effects on the two PD parameters: Cognitive load reduced the participants’ utilitarian inclinations, but left their deontological inclinations unaffected. In contrast, induced empathic concern increased deontological inclinations but had no effect on utilitarian inclinations. Furthermore, the two PD parameters were found to be only weakly correlated across participants in one study (*r* = 0.28), and not significantly correlated in two other studies, supporting the assumption that the processes underlying D and U are largely independent. A later meta-analysis of the PD parameter correlations obtained in 40 moral judgment studies corroborated this conclusion (meta-analytic *r* = 0.10; Friesdorf et al., 2015).

Following Conway and Gawronski’s (2013) article, the PD procedure has been used in numerous other experiments to clarify the effects of diverse variables on deontological and utilitarian inclinations in moral dilemmas. Like the original study, several of these studies obtained evidence for the importance of affective processes in moral dilemmas. For example, Lee and Gino (2015) found that the instruction to suppress one’s emotional reactions to moral dilemmas decreased deontological but not utilitarian inclinations; and Reynolds and Conway (2018) found that (a) manipulating the aversiveness of the harmful action increased deontological inclinations but decreased utilitarian inclinations, whereas (b) manipulating the aversiveness of the outcome increased both inclinations (see also Christov-Moore et al., 2017, for a related brain-imaging study). Other PD work has helped to clarify how moral judgments are influenced by social considerations (Rom and Conway, 2018), feelings of power (Fleischmann et al., 2019), self-awareness (Reynolds et al., 2019), distrust (Conway et al., 2018b), analytical thinking style (Li et al., 2018), gender (Friesdorf et al., 2015; Armstrong et al., 2019), and the presentation of moral dilemmas in a foreign language (Hayakawa et al., 2017; Muda et al., 2018).

Apart from demonstrating that the PD procedure can detect patterns of deontological and utilitarian inclinations that remained invisible in earlier moral dilemma research, which relied on a one-dimensional measure of moral inclinations (the percentage of utilitarian responses), the PD studies have provided evidence that the U parameter indeed measures, at least primarily, the person’s tendency to maximize the overall benefit (e.g., Conway and Gawronski, 2013; Conway et al., 2018a; see also Patil et al., 2020). By contrast, the interpretation of the D parameter is somewhat less clear. Although, as mentioned, there is evidence supporting the assumption that the D parameter reflects affective aversions to harmful actions (e.g., Conway and Gawronski, 2013; Lee and Gino, 2015), other evidence suggests that the D parameter can also reflect the person’s explicit consideration of deontological moral principles (Gamez-Djokic and Molden, 2016). For this reason, the two PD parameters cannot be interpreted as precisely reflecting the processes assumed to underlie these inclinations in the original dual-process model (Greene et al., 2001) or its more recent variants. Rather, the two parameters seem to reflect the joint effect of several different processes that coalesce into the two conflicting motivational tendencies that proximately underlie judgment and decision making in moral dilemmas. Still, we agree with Conway et al.’s (2018a, p. 244) conclusion that “although other processes not modeled [in the PD procedure] may play a role, ultimately deontological responses seem to reflect relatively more affective processing centered on harmful actions, whereas utilitarian responses appear to reflect relatively more deliberative reasoning centered on outcomes.”

## Present Research

As mentioned, the effects of instrumentality and personal force on moral judgments in dilemma situations are well supported; however, so far it is not entirely clear whether these factors influence primarily deontological inclinations, utilitarian inclinations, or both to a similar degree. To illustrate the possibilities, consider again the car accident scenario described above, and compare the personal version of this scenario (hit the old lady directly) to the impersonal version (hit a scaffold which by collapsing kills the lady). Based on previous research (e.g., Conway and Gawronski, 2013), one can predict that swerving will be considered more appropriate in the impersonal scenario. But why? There are at least three different combinations of deontological and utilitarian inclinations that predict this pattern of judgments. First, the personal scenario could elicit a stronger deontological inclination than the impersonal scenario, which then results in a higher rate of harm-rejecting responses. Second, the impersonal scenario could evoke a stronger utilitarian inclination than the personal scenario, which results in higher rates of harm-approval. Third, both inclinations could be simultaneously affected by personal force in the described way, and the moral judgments reflect the joint effect of these counteracting forces. Analogous possibilities exist with regard to the effects of the instrumentality of the harm on moral judgments.

To clarify the relative importance of deontological and utilitarian inclinations for the effects of instrumentality and personal force on moral judgment, three experiments were conducted. Experiment 1 examined the effect of instrumentality of the harm (harm as means vs. harm as side-effect) on deontological and utilitarian inclinations. Experiment 2 added personal force (personal vs. impersonal moral dilemmas) as a second important dilemma conceptualization variable. Again, the aim was to clarify to which degree this factor influences deontological and utilitarian inclinations. In addition, this experiment allowed us to re-examine the effects of the interaction of the two variables (as found in earlier work, see Greene et al., 2009). Experiment 3 was a pre-registered replication of Experiment 2 that was conducted to enhance the confidence in the findings of Experiments 1 and 2 by replicating their results with a much larger sample. The present research can be considered as a “far conceptual replication” of the study by Greene et al. (2009) in the sense of LeBel et al. (2018): It manipulated the same variables, but used a different set of moral dilemmas, and focused on different dependent variables (the PD parameters).

Before proceeding, it should be noted that in our experiments, the PD parameters were derived from the participants’ answers to the question whether they themselves would perform the harm-causing action. Following Christensen et al. (2014) and others (e.g., Koenigs et al., 2007; Bartels, 2008), we focused on moral choice since this is ultimately what matters most in moral dilemmas (see also Foot, 1967; Waldmann and Dieterich, 2007). However, in many moral dilemma studies, the participants were instead asked to judge the moral appropriateness of the harmful action, either from the first-person perspective (“Is it appropriate for you to do X?” e.g., Conway and Gawronski, 2013), or from the third-person perspective (“Is it morally acceptable for Joe to do X?” e.g., Greene et al., 2009). As pointed out by several researchers, these different dependent variables cannot simply be regarded as equivalent (e.g., Tassy et al., 2013; Christensen et al., 2014). We will return to this question in the *General Discussion*, after presenting our results obtained for moral choice.

### Hypotheses

Table 1 contains an overview of the hypotheses (as well as the findings) of Experiments 1–3.

**TABLE 1:** Overview of the hypotheses tested and the results obtained in Experiments 1, 2, and 3.

Hypothesis	Experiment 1	Experiment 2	Experiment 3
**H1a.** The PD Deontology parameter is higher when harm is a means than when harm is a side-effect.	Confirmed	Confirmed	Confirmed
**H1b.** The PD Utilitarianism parameter is unaffected by the instrumentality manipulation.	Rejected	Rejected	Rejected

**H2a.** The PD Deontology parameter is higher in personal than in impersonal dilemmas.	Not tested	Confirmed	Confirmed
**H2b.** The PD Utilitarianism parameter is higher in impersonal than in personal dilemmas.	Not tested	Confirmed	Confirmed

**H3.** There is an interaction effect of instrumentality and personal force on deontological inclinations, utilitarian inclinations, or both.	Not tested	Supported	Not supported

**H4a.** The individual PD Deontology and PD Utilitarianism parameters are largely independent overall, and in particular are not strongly negatively correlated.	Confirmed	Confirmed	Confirmed
**H4b.** The correlation between the PD parameters differs between experimental conditions.	Not supported	Not supported	Confirmed

Concerning the dilemma conceptualization variable *instrumentality of the harm* (means vs. side-effect), we expected to replicate the typical finding of previous studies, that harm approval rates are higher in side-effect dilemmas than in means dilemmas. Our main interest, however, was in the processes underlying this response pattern. To explain the effects of instrumentality, Greene (2014) proposed an extension of the dual-process model called the *modular myopia hypothesis*, which specifies when affective processes dominate over deliberative processes. According to this hypothesis, an evolutionary “action inspector module” monitors action plans and evokes a negative emotion whenever harm is detected as a means to achieving a desirable end, but not if it the harm is an unintended (even if foreseen) side-effect of the action (Greene, 2014; see also Cushman, 2014). This hypothesis predicts stronger deontological inclinations in moral dilemmas depicting harm as a means than in side-effect dilemmas. In contrast, utilitarian inclinations should not be affected by the instrumentality manipulation, because whether the harm is intended or is a mere side-effect is presumably irrelevant for outcome-maximization. Based on the assumption that deontological and utilitarian inclinations are reflected in the PD Deontology and PD Utilitarianism parameters, respectively, we therefore predicted (see Hypothesis H1 in Table 1), that the D parameter would be higher for harm as means than for harm as side-effect, whereas the U parameter would be unaffected by the instrumentality manipulation.

With regard to the manipulation of *personal force*, previous work suggests that personal dilemmas elicit stronger affective reactions than impersonal dilemmas, whereas impersonal dilemmas are more likely to evoke deliberative processes than personal dilemmas (Greene et al., 2001, 2004). Based on these findings, we predicted that the D parameter would be higher for personal than for impersonal dilemmas, whereas the U parameter would be higher for impersonal than for personal dilemmas (see Table 1).

Greene et al. (2009) additionally found that the instrumentality of the harm affected moral judgments only in personal dilemmas, i.e., if personal force was present. Under the assumption that moral judgments are based on deontological and/or utilitarian inclinations, this finding suggests that a similar interaction will be obtained for the PD parameters of one or both of these inclinations. However, the exact nature of these interactions was difficult to predict. Therefore, we refrained from speculating and only predicted the existence of an interaction of instrumentality and personal force for one or both of the two PD parameters (Hypothesis H3, Table 1).

Finally, we formulated two hypotheses (H4a and H4b, see Table 1) between the correlations about the two PD parameters. H4a states that overall (i.e., across experimental conditions), the D and U parameters would be unrelated, or only moderately correlated. This hypothesis simply predicts the successful replication of findings from previous experiments using the PD procedure (see the meta-analysis of Friesdorf et al., 2015). However, the findings of Friesdorf et al. (2015) still allow for the possibility that the PD parameters correlate more strongly in specific situations, not systematically considered in the dilemmas included in their meta-analysis. Specifically, a reflection on the nature of the experimental manipulations of instrumentality and personal force suggested to us that these manipulations might not only affect the relative contributions of deontological and utilitarian inclinations to moral judgment, but also their relation to one another. That is, we suspected that the situational context could be a moderator of the PD parameter correlations. This lead to the hypothesis (H4b) that the correlation between the PD parameters would differ between experimental conditions.

## Experiment 1

In Experiment 1, we used the PD procedure (Conway and Gawronski, 2013) to disentangle the effects of the instrumentality of the harm (i.e., harm as a means vs. harm as a side-effect) on deontological and utilitarian inclinations in moral dilemmas. As mentioned, the instrumentality of the harm has been found to exert a strong effect on the judgment of harmful actions in moral dilemmas (O’Neill and Petrinovich, 1998; Hauser et al., 2007; Greene et al., 2009; Cushman, 2014). However, it is still unknown to which degree instrumentality affects deontological inclinations, utilitarian inclinations, or both.

### Method

#### Participants

A target sample size of *N* = 60 was determined prior to conducting the experiment. Data collection was not continued after reaching the target sample size. Sixty-one students (50 female) participated in exchange for course credit (*M*_age_ = 21.72, *SD* = 5.04). A sensitivity power analysis for a 2 (within, parameter type: PD Deontology vs. PD Utilitarianism) × 2 (between, instrumentality: means vs. side-effect) repeated measures ANOVA, conducted using G^∗^Power (Faul et al., 2007) indicated a minimum detectable effect size of $\eta_{\text{p}}^{2}$ = 0.088, assuming α = 0.05, 1–β = 0.80, and a correlation of *r* = −0.45 between the repeated measures (i.e., the PD parameters), corresponding to the actually obtained correlation in this study. The detectable effect size corresponds to the typical effects of the experimental manipulations used in previous moral judgment PD studies (e.g., Conway and Gawronski, 2013, Study 2). However, we ultimately decided to analyze the data using logistic regression, which is more adequate for our dependent variables (the PD parameters)^1^.

#### Materials

A set of 20 moral dilemmas, comprising ten dilemmas for harm as means and ten for harm as side-effect, was compiled from the literature (Cushman et al., 2006; Conway and Gawronski, 2013; Christensen et al., 2014). In the base version of the scenarios, the harm-rejection tendency (deontological inclination) and the outcome-maximization tendency (utilitarian inclination) suggested diverging responses. Following Conway and Gawronski (2013), we constructed, for each of these *incongruent* scenarios, a parallel *congruent* version in which the deontological and utilitarian inclinations favored the same response. Incongruent moral dilemmas were defined as situations in which the overall consequences of acting (i.e., performing a harmful action) were more beneficial than those of not acting (Conway and Gawronski, 2013). Congruent dilemmas were defined as situations in which the positive effects of performing the harmful action were minimized, such that both deontological and utilitarian inclinations suggested the rejection of the harmful action.

Altogether, there were 40 dilemmas, 20 incongruent, and 20 congruent ones. All scenarios were matched on grammatical structure. Instrumentality of the harm was experimentally manipulated, whereas other dilemma conceptualization variables that have been found to influence judgments and decisions in previous studies (see Christensen et al., 2014) were either held constant (personal force) or counter-balanced (benefit recipient, evitability). The complete materials are available in the Supplementary Material and at https://osf.io/zxykm/.

#### Procedure

The experiment was conducted in a computer lab of the Institute of Psychology at the University of Greifswald. Up to four participants were invited for group sessions. They worked on separate computers shielded by room dividers. The participants were randomly assigned to one of two conditions (means vs. side-effect), with each group responding to 20 moral dilemmas, ten incongruent and ten corresponding congruent ones. The experiment was programmed using *OpenSesame* (Mathôt et al., 2012).

In each trial, one of the dilemmas was presented on the monitor, followed by the question “Do you [action verb] so that [consequence]?” e.g., “Do you swerve and hit the old lady to avoid hitting the young mother and child?” (Christensen et al., 2014; *Present Research*). The participants responded to the moral dilemmas by clicking on one of two response buttons, labeled “yes” and “no,” presented below the question. Participants were randomly assigned to one of two button orders (“yes” left and “no” right, or the reverse). To counteract sequence and habituation effects, five different semi-random orders of the scenarios were created for each condition. These orders were random with two exceptions: (a) no more than three congruent or incongruent trials occurred consecutively; (b) at least six other dilemmas were presented between an incongruent dilemma and its corresponding congruent version.

### Results

The participants judged the harmful action as acceptable in 14% (*SD* = 13) of the congruent and in 55% (*SD* = 29) of the incongruent dilemmas. A logit mixed-effects model analysis (see below for more explanation) for scenario type (congruent/incongruent) as a repeated measures factor showed this difference to be significant, χ(1) = 73.43, *p* < 0.001, *OR* = 9.28. The harm approval rates were similar to those found in previous studies (Conway and Gawronski, 2013), despite the fact that we used a different question format (first-person choice rather than third-person evaluation).

Parameter estimates for deontological inclination (D) and utilitarian inclination (U) were caclulated for each participant based on equations derived from the processing tree (Figure 1; see also Jacoby, 1991; Conway et al., 2018a). Hypotheses H1 and H4, the two hypotheses that could be tested in Experiment 1, were then evaluated using these parameters as dependent variables. Descriptive statistics of the PD parameters obtained in Experiments 1–3 are shown in Table 2.

**TABLE 2:** Means and standard deviations (in parentheses) of the conventional measure of moral inclinations and the Process Dissociation (PD) parameters for deontological inclinations (PD Deontology) and utilitarian inclinations (PD Utilitarianism) in Experiments 1–3.

		Impersonal		Personal	
		Side-effect	Means	Side-effect	Means
Experiment 1	Conventional measure			0.71 (0.20)	0.39 (0.28)
	PD Deontology (D)			0.62 (0.27)	0.80 (0.26)
	PD Utilitarianism (U)			0.55 (0.20)	0.28 (0.22)
	Parameter correlation			−0.20 (*p* = 0.285)	−0.48 (*p* = 0.007)
Experiment 2	Conventional measure	0.73 (0.24)	0.44 (0.22)	0.55 (0.29)	0.41 (0.25)
	PD Deontology (D)	0.47 (0.28)	0.71 (0.22)	0.67 (0.25)	0.79 (0.22)
	PD Utilitarianism (U)	0.50 (0.21)	0.23 (0.14)	0.35 (0.25)	0.28 (0.19)
	Parameter correlation	−0.20 (*p* = 0.374)	−0.11 (*p* = 0.633)	−0.06 (*p* = 0.789)	−0.37 (*p* = 0.088)
Experiment 3	Conventional measure	0.78 (0.17)	0.56 (0.26)	0.66 (0.24)	0.41 (0.26)
	PD Deontology (D)	0.42 (0.28)	0.56 (0.27)	0.55 (0.30)	0.76 (0.22)
	PD Utilitarianism (U)	0.50 (0.24)	0.25 (0.21)	0.42 (0.25)	0.25 (0.20)
	Parameter correlation	−0.16 (*p* = 0.182)	−0.24 (*p* = 0.041)	−0.24 (*p* = 0.036)	−0.45 (*p* < 0.001)

*The correlations between the parameters are also shown.*

In most previous moral dilemma studies that used the PD procedure, the parameters were analyzed by means of ANOVA after being standardized (*z*-transformation; e.g., Conway and Gawronski, 2013; Lee and Gino, 2015). However, because the parameter values, being probabilities (Jacoby, 1991), are constrained to [0,1], their errors are not normally distributed, and their variance depends on the mean, a logistic regression (logit) model is better suited for their analysis (Jaeger, 2008)^2^. Because parameter type was included as a within-subjects factor in the analyses, a logit mixed-effect analysis was conducted, using the glmer function from the lme4 package (Bates et al., 2015) in R (R Core Team, 2018). The dependent variable consisted of the weighted raw parameter scores, using 20 as the weight because the parameters were derived from responses to 20 dilemmas.

The independent variables were parameter type (D vs. U) and instrumentality (means vs. side-effect), with a random intercept for participants and a by-subject random slope for the effect of parameter type. To be suitable for the logit analysis the dependent variable had to be slightly conditioned as follows: First, negative parameter values, if they occurred, were set to 0 (there were no such values in Experiment 1, but a few occurred in Experiments 2 and 3 for the U parameter). Negative parameter estimates are meaningless for probabilities, but can occur with errorful data. Setting these estimates to 0 means to set them to the closest theoretically meaningful value (see Rouder et al., 2008). Second, to avoid fitting problems, the parameter values were rounded to yield integer values when weighted by 20 (e.g., 0.42 was rounded to 0.40 and 0.44 to 0.45). The significance of the fixed effects was evaluated with likelihood ratio tests (LRT), by comparing a model including the effect to a model without it. Because the LRT in mixed-effects logit analyses can be unreliable for small to moderate samples (Li and Redden, 2015), we also consulted the Wald *z*-tests for the parameter estimates (see Table 3); however, the results of the two tests were always consistent. The odds ratio (OR) was used as the effect size index.

**TABLE 3:** Results of the logit mixed-effect model analysis for Experiment 1.

	*b* (SE)	*z* (*p*)	Odds ratio (0.95 CI)
Intercept	0.79 (0.41)		
Parameter (U)	−0.60(0.51)	−1.17(0.241)	0.55(0.20,1.49)
Instrumentality (means)	1.66^∗∗^(0.61)	2.73 (0.006)	5.29(1.60,17.46)
Parameter (U) × Instrumentality (means)	−3.05^∗∗∗^(0.75)	−4.05 (< 0.001)	0.05(0.01,0.21)

***p < 0.001, **p < 0.01, *p < 0.05, based on the Wald z statistic.

**FIGURE 2:**
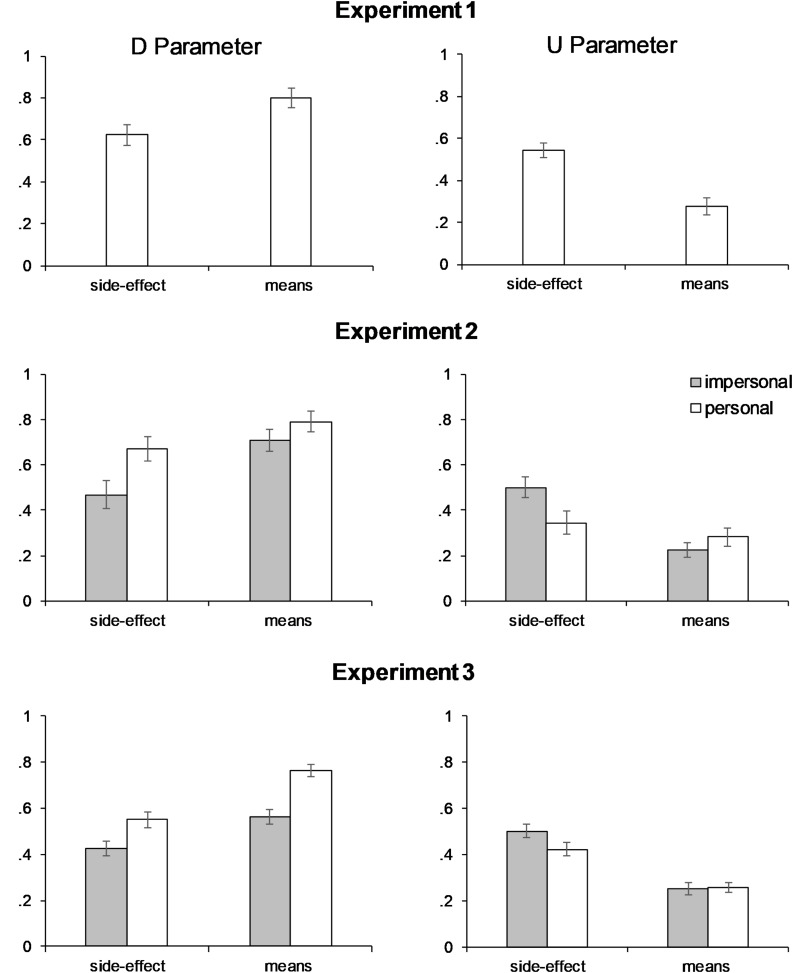
Means of the PD Deontology and Utilitarianism parameters in Experiments 1-3. Error bars indicate the standard error of the mean.

Consistent with H1, the two-way interaction of parameter type and instrumentality in the mixed logit analysis was significant, χ(1) = 14.98, *p* < 0.001, *OR* = 0.05 (see Table 2 for descriptive statistics and Table 3 for the parameter estimates and the associated Wald tests). This interaction was followed up by separate logit analyses for the two parameters, using the glm function of R. Because there was evidence for overdispersion in the data, the quasibiomial rather than the binomial distribution was specified in these analyses (see Crawley, 2012). The logit analyses confirmed that, consistent with H1a, the D parameter was significantly higher in the means (*M* = 0.80) condition than in the side-effect condition (*M* = 0.62), χ(1) = 7.04, *p* = 0.008, *OR* = 2.49 (see also Figure 2). Contradicting H1b, however, the U parameter was signficantly higher in the side-effect condition (*M* = 0.55) than in the means condition (*M* = 0.28), χ(1) = 22.62, *p* < 0.001, *OR* = 0.32 (see Figure 2). Hence, the instrumentality of the harm affected both the D and U parameters, and in opposite directions.

Because Friesdorf et al. (2015) had found that female participants have higher D scores than males, we conducted another mixed-effects logit analysis that included sex and the interaction of sex with parameter type as additional predictors. No significant main effect of sex was found, χ(1) = 0.02, *p* = 0.884, but there was an interaction of sex and parameter type, χ(2) = 3.95, *p* = 0.047, *OR* = 6.34. Consistent with the findings of Friesdorf et al. (2015), males (*M* = 0.61) had lower D parameters than females (*M* = 0.73), χ(2) = 12.88, *p* < 0.001, *OR* = 0.57. In addition, the U parameter was significantly higher for males (*M* = 0.47) than for females (*M* = 0.40), χ(2) = 3.90, *p* = 0.048, *OR* = 1.34.

To compare the results of the PD analysis to those obtained with the conventional measure of moral inclinations, we also analyzed the relative frequency of utilitarian judgments in incongruent dilemmas. According to the logic underlying the conventional measure, high scores reflect the predominance of utilitarian and low scores the predominance of deontological inclinations. Consistent with previous research, a logit analysis (with weights set to 10 because the relative frequencies were based on the responses to the 10 incongruent dilemmas only) indicated that the proportion of utilitarian judgments was lower in the means condition (*M* = 0.39) than in the side-effect condition (*M* = 0.71), χ(1) = 61.50, *p* < 0.001, *OR* = 0.27.

To test H4, we computed the correlations between the participants’ PD parameters for deontological and utilitarian inclinations. A significant negative correlation of *r* = −0.45, *p* < 0.001, was found, contradicting the assumption that the two parameters are completely independent. However, still in agreement with H4a, the size of the correlation was moderate (20% explained variance). Contradicting H4b, the size of the correlation did not differ significantly between the two experimental conditions (*r*_side–effect_ = −0.20, *p* = 0.285 vs. *r*_means_ = −0.48, *p* = 0.007; *z* = 1.19, *p* = 0.235).

### Discussion

Experiment 1 tested two of the four hypotheses described in the introduction, H1 and H4. Hypothesis 1 was that the PD Deontology parameter would be higher for harm as means than for harm as side-effect (H1a), whereas the PD Utilitarianism parameter would be unaffected by the instrumentality manipulation (H1b). Whereas H1a was supported by the results, H1b was disconfirmed: The instrumentality manipulation affected both deontological and utilitarian inclinations. In fact, the effect of instrumentality on U was descriptively stronger than its effect on D (see Figure 2): Expressed in terms of the raw parameter scores, U decreased by 0.27 in the means condition, relative to the side-effect condition, whereas D increased by 0.18 in the means relative to the side-effect condition.

The finding that the instrumentality manipulation affected both deontological and utilitarian inclinations constitutes a challenge to the modular myopia hypothesis (Greene, 2014), but it is compatible with “soft” versions of the dual-process model of moral judgment (e.g., Skitka and Conway, 2019), according to which affective and deliberative processes can influence both deontological and utilitarian inclinations.

Consistent with Friesdorf et al. (2015), females had on average a higher D parameter than males, and we also found higher U parameters for males than for females.

Hypothesis 4a was that the D and U parameters are statistically largely independent overall, and in particular are not strongly negatively related. Partly contradicting the independence hypothesis, there was a significant negative correlation between D and U, indicating that higher D scores were associated with lower U scores and vice versa. Still, the size of the overall correlation was modest (*r* = −0.45) and low in one of the two experimental conditions (*r*_side–effect_ = −0.20). Hence, if judged by the size of the obtained correlations, H4a was broadly supported. Certainly the obtained size of the negative correlations is too low to justify the conventional measure of moral inclinations.

Hypothesis 4b predicted significantly different correlations of the PD parameters in the two experimental conditions. This hypothesis was not statistically supported. However, given the sizable differences between the correlations obtained in the two conditions, this negative finding may have been due to insufficient statistical power.

Analogous to some previous PD studies of moral judgment (e.g., Reynolds and Conway, 2018), the PD procedure revealed that a dilemma conceptualization variable can have opposite effects on deontological and ulilitarian inclinations, a pattern the conventional measure of moral inclinations is incapable of detecting. This finding constitutes additional support for the PD measurement of moral inclinations.

## Experiment 2

Experiment 2 had two aims. The first aim was to replicate the unpredicted finding of Experiment 1, that the manipulation of instrumentality affected not only deontological but also utilitarian inclinations. The second aim was to investigate the effects of a second important dilemma conceptualization variable, personal force, on deontological and utilitarian inclinations.

### Method

#### Participants

A target sample size of *N* = 90 was determined prior to data collection for the initially planned ANOVA design, as being sufficient to detect effects of the size found in Experiment 1 with a power of 0.80 (the minimum detectable effect size for *N* = 90 is η^2^ = 0.077). For details of the power analysis, see the online Supplementary Material to this article.

Ninety students (68 female; *M*_age_ = 23.96, *SD* = 6.33) at the University of Greifswald participated in exchange for course credit (*n* = 50) or a compensation of €5 (*n* = 40). To assure independence of the samples of Experiments 1 and 2, students who had already participated in Experiment 1 were not allowed to take part in Experiment 2. Two participants were excluded from the data analyses because they failed to comply with the instructions (as suggested by extremely short decision times, they responded without reading the dilemmas). Results are thus based on a sample size of *N* = 88. Data collection was not continued after reaching the target sample size.

#### Materials

Because personal force was an additional between-subjects factor in Experiment 2, an impersonal dilemma version had to be created for each of the 40 personal scenarios used in Experiment 1. This resulted in 80 scenarios. Whereas the personal dilemmas described an actor who directly causes a harmful outcome in the attempt to prevent greater harm, the impersonal dilemmas described an actor who initiates a process which then, by its own dynamics, causes the harmful outcome. For example, in the personal version of the car accident dilemma, it was only possible to save the mother and her child by steering the car toward, and thereby injuring or killing, the old lady; whereas in the impersonal version of the scenario, it was only possible to save the mother and her child by swerving into a scaffold that, by collapsing, will injure or kill the old lady. All materials are available at https://osf.io/zxykm/ (see also Supplementary Material).

#### Procedure

The procedure corresponded to that of Experiment 1. The only difference was that in Experiment 2 there were four conditions, which resulted from crossing the factors instrumentality and personal force (personal/means, personal/side-effect, impersonal/means, and impersonal/side-effect). Participants were randomly assigned to one of the four conditions.

### Results

The participants accepted the harmful action in 21% (*SD* = 17) of the congruent and in 53% (*SD* = 28) of the incongruent scenarios, χ(1) = 76.83, *p* < 0.001, *OR* = 5.28. These harm approval rates are again very similar to those found in previous comparable studies (Conway and Gawronski, 2013). Parameters for deontological and utilitarian inclinations were computed for each participant as described in the *Method* section of Experiment 1. Table 2 shows the PD parameter means and standard deviations (see also Figure 2), and parameter correlations.

A logit mixed-effects model was fitted to the raw parameter values after setting five negative parameter estimates to 0 (see Experiment 1 for an explanation). The independent variables were parameter type (D vs. U), instrumentality (means vs. side-effect), and personal force (personal vs. impersonal). A random intercept was specified for participants and a by-subject random slope for the effect of parameter type. Parameter estimates and associated statistics are shown in Table 4. Separate logit analyses were run to follow up on the obtained interactions of instrumentality and personal force with parameter type.

**TABLE 4:** Results of the logit mixed-effect model analyses for Experiments 2–3.

	Experiment 2			Experiment 3		
	*b* (SE)	*z* (*p*)	Odds ratio (0.95 CI)	*b* (SE)	*z* (*p*)	Odds ratio (0.95 CI)
Intercept	−0.19 (0.33)			−0.52* (0.20)		
Parameter (U)	0.15 (0.46)	0.32 (0.747)	1.16 [0.47, 2.83]	0.49 (0.29)	1.73 (0.084)	1.64 [0.94, 2.87]
Instrumentality (means)	1.42** (0.48)	2.98 (0.003)	4.13 [1.63, 10.48]	0.88** (0.29)	3.07 (0.002)	2.41 [1.37, 4.22]
Personal Force (personal)	1.20* (0.47)	2.54 (0.011)	3.33 [1.31, 8.44]	0.78** (0.29)	2.75 (0.006)	2.20 [1.25, 3.84]
Parameter (U) × Instrumentality (means)	−2.78*** (0.65)	−4.28 (< 0.001)	0.06 [0.02, 0.22]	−2.28*** (0.40)	−5.62 (< 0.001)	0.10 [0.05, 0.23]
Parameter (U) × Personal Force (personal)	−1.99** (0.65)	−3.08 (0.002)	0.14 [0.04, 0.49]	−1.22** (0.40)	−3.03 (0.002)	0.30 [0.13, 0.65]
Instrumentality (means) × Personal Force (personal)	−0.45 (0.68)	−0.67 (0.503)	0.63 [0.17, 2.40]	0.55 (0.41)	1.33 (0.183)	1.73 [0.77, 3.85]
Parameter (U) × Instrumentality (means) × Personal Force (personal)	1.51 (0.93)	1.63 (0.104)	4.51 [0.73, 27.64]	−0.07 (0.58)	−0.13 (0.898)	0.93 [0.30, 2.88]

***p < 0.001, **p < 0.01, *p < 0.05, based on the Wald z statistic.

In contrast to the first experiment, Experiment 2 allowed to test all four hypotheses described in the *Introduction*. Concerning the effects of instrumentality (H1), the logit mixed-effects model revealed a significant two-way interaction of parameter type and instrumentality, χ(1) = 21.25, *p* < 0.001, *OR* = 0.06. In line with H1a and the findings of Experiment 1, the D parameter was significantly higher in the means conditions (*M* = 0.75) than in the side-effect conditions (*M* = 0.57; see Figure 2 and Table 4). Contradicting H1b, but again replicating Experiment 1, the U parameter was significantly higher in the side-effect conditions (*M* = 0.42) than in the means conditions (*M* = 0.25). Separate logit analyses for the two PD parameters with instrumentality and personal force as the independent variables were conducted to verify this pattern. Again the quasibinomial rather than the binomial distribution was used in these analyses because there was evidence for overdispersion. The results confirmed that D was higher in the means than in side-effect conditions, χ(1) = 9.46, *p* = 0.002, *OR* = 2.80, whereas U was lower in the means than in the side-effect conditions, χ(1) = 19.03, *p* < 0.001, *OR* = 0.30.

Hypothesis 2 also received support: There was a significant two-way interaction of parameter type and personal force, χ(1) = 6.85, *p* = 0.009, *OR* = 0.14 (see Figure 2 and Table 4). Separate logit regressions for each parameter confirmed that, in line with H2a, the PD Deontology parameter was significantly higher in personal (*M* = 0.73) than impersonal dilemmas (*M* = 0.59), χ(1) = 6.56, *p* = 0.010, *OR* = 2.32. Also as predicted (H2b), the reverse was found for the PD Utilitarianism parameter (*M* = 0.31 and *M* = 0.36, for U in personal and impersonal dilemmas, respectively), χ(1) = 5.82, *p* = 0.016, *OR* = 0.53.

In addition, there was support for hypothesis H3: The separate logit analyses for D and U detected a significant interaction of instrumentality and personal force on U, χ(1) = 5.25, *p* = 0.022, *OR* = 2.50. No significant interaction effect was found for D, χ(1) = 0.62, *p* = 0.432. Follow-up analyses (separately for the means and side-effect dilemmas) revealed that the predicted effect of personal force on U (a lower U in personal than impersonal dilemmas) occurred in the side-effect scenarios, χ(1) = 4.79, *p* = 0.029, *OR* = 0.53, but not in the means scenarios, χ(1) = 1.08, *p* = 0.298.

Again, we conducted an additional analysis that also included included sex and the interaction of sex and parameter type as predictors, to test for possible sex differences. No significant main effect of sex, χ(1) = 0.63, *p* = 0.427, and no interaction with parameter type was found, χ(1) = 0.79, *p* = 0.373. Both parameters were nearly identical for females and males (D: *M*_female_ = 0.65, *M*_male_ = 0.69; U: *M*_female_ = 0.34, and *M*_male_ = 0.28). Analogous to Experiment 1, we also conducted an analysis for the conventional measure of moral inclinations, the relative frequency of utilitarian judgments. Means and standard deviations for the conventional measure are shown in Table 2.

Consistent with the familiar pattern of rejecting the harmful action in means and personal scenarios, but accepting it in side-effect and impersonal scenarios, the frequency of utilitarian judgments was highest in the impersonal/side-effect condition and lowest in the personal/means condition (*M*s = 0.73 and 0.41, respectively). A logit analysis with instrumentality and personal force as independent variables found significant main effects for instrumentality, χ(1) = 41.18, *p* < 0.001, *OR* = 0.28, and personal force, χ(1) = 11.06, *p* < 0.001, O*R* = 0.43, in the expected direction. In addition, a significant interaction of instrumentality and personal force was obtained, χ(1) = 6.86, *p* = 0.009, *OR* = 2.08, indicating that high personal force reduced utilitarian judgments in side-effect dilemmas, χ(1) = 17.58, *p* < 0.001, *OR* = 0.43, but not in means dilemmas, χ(1) = 0.33, *p* = 0.563.

Hypothesis 4a was partially supported: Although, as in Experiment 1, there was a significant negative correlation between the PD parameters, this correlation was modest, *r* = −0.29, *p* = 0.006, and well within the range of previously obtained PD parameter correlations (Friesdorf et al., 2015). The second part of the hypothesis (H4b) was again unsupported: Even though the PD parameter correlations differed considerably between the experimental conditions (see Table 2), pairwise comparisons revealed that none of these differences were statistically significant (all *z*s ≤ 1.012, all *p*s ≥ 0.156).

## Experiment 3

To enhance confidence in the findings of Experiments 1 and 2, we conducted a third experiment with a much larger sample size. The materials and the experimental procedure of Experiment 3 were the same as in Experiment 2, with the exception of slight changes required by the online presentation of the study, plus a number of improvements to the German translations of the original English scenarios. These changes were made to enhance the clarity of the dilemmas. For example, overly long sentences were divided into several shorter ones, a few words were exchanged, and the punctuation and orthography was revisited. Furthermore, different from Experiments 1 and 2, Experiment 3 was pre-registered, i.e., all hypotheses (which corresponded to those described in the *Present Research* section), procedures, and a sampling and analysis plan were registered prior to data collection. The pre-registration is available at https://osf.io/zxykm/.

### Method

#### Participants

A target sample size of *N* = 300 was decided on prior to data collection (see Supplementary Material). For the originally planned ANOVA analysis, this sample size is sufficient to detect an effect size of η^2^ = 0.023 with a power of 0.80, which is a much smaller effect than the effects obtained in Experiments 1 and 2 and in previous comparable studies (e.g., Conway and Gawronski, 2013). Three hundred and ten participants were recruited for the online study at two German University campuses. Participation in the study was compensated with course credit or the opportunity to take part in a raffle in which one of fifteen 50-Euro Amazon vouchers could be won. Two participants informed us that they had mistakenly taken part in the study twice; the duplicate entries were removed prior to the data analyses. A preliminary analysis of total processing time revealed ten outliers (> 3 *SD* below or above the mean of the logarithmized processing time), i.e., participants who responded either very quickly (in one case, in less than 15 s) or very slowly (several hours). These outliers were also excluded from the analyses^3^. All analyses are thus based on a sample size of *N* = 300 (69 males, three indicated a gender other than female or male; *M*_age_ = 24.03 years, *SD* = 5.25).

#### Materials and Procedure

The materials and procedure were essentially the same as in Experiment 2, the main difference being that the experiment was conducted as an online study. To this end, Experiment 2 was re-implemented as a web experiment using *SoSciSurvey*, a browser-based generator for online surveys and web experiments (www.soscisurvey.de, Munich, Germany). The order of presentation of the dilemmas was determined using the same randomization procedure as in Experiments 1 and 2. Minor changes were made to the on-screen appearance of the moral dilemmas (e.g., bold fonts and response buttons) to support the online presentation of the materials. All materials are available in the online supplement to this article and at https://osf.io/zxykm/. The complete web experiment is available on request from the first author.

### Results

The harmful action was accepted in 25% (*SD* = 20) of the congruent scenarios and in 60% (*SD* = 27) of the incongruent scenarios. This difference was significant, χ(1) = 295.00, *p* < 0.001, *OR* = 5.99, and is comparable in size to that found in Experiments 1 and 2 and in prior studies (e.g., Conway and Gawronski, 2013). PD Parameters for deontological and utilitarian inclinations were computed for each participant as in Experiments 1 and 2. The means of the PD parameters (after 11 negative values of U had been set to 0) and the parameter correlations in the different conditions are shown in Table 2, and the means are also displayed in Figure 2.

The same analysis strategy as in Experiment 2 was used: A logit mixed-effects regression with parameter type, instrumentality, and personal force as independent variables, followed by separate logit analyses for each parameter. These analyses replicated the findings of the previous experiments. Table 4 reports the parameter estimates obtained in the mixed-effects regression.

With regard to H1, the analysis revealed a significant interaction of parameter type and instrumentality, χ(1) = 64.08, *p* < 0.001, *OR* = 0.10, that was consistent with the findings of Experiments 1 and 2. As confirmed by separate logit analyses for each parameter, the D parameter was significantly higher in the means conditions (*M* = 0.66) than in the side-effect conditions (*M* = 0.49), χ(1) = 9.97, *p* = 0.002, *OR* = 1.78, whereas the U parameter was significantly lower in the means (*M* = 0.25) than in the side-effect conditions (*M* = 0.46), χ(1) = 42.56, *p* < 0.001, *OR* = 0.34. (As in Experiment 2, the quasibinomial rather than the binomial distribution was specified in these analyses to account for overdispersion). These results corroborate the findings of Experiments 1 and 2, and again contradict the prediction that we derived from the modular myopia hypothesis, that utilitarian inclinations will remain unaffected by the manipulation of instrumentality.

Regarding the personal force manipulation (H2), there was a significant two-way interaction of parameter type and personal force, χ(1) = 18.86, *p* < 0.001, *OR* = 0.30 (see Table 4). Separate logit regressions found that the D parameter was significantly higher in personal (*M* = 0.65) than in impersonal dilemmas (*M* = 0.49), χ(1) = 8.03, *p* = 0.005, *OR* = 1.68, which is consistent with H2a and with the findings of Experiment 2. Also in agreement with the findings of Experiment 2 and with H2b, the U parameter was lower in personal (*M* = 0.34) than impersonal dilemmas (*M* = 0.38), χ(1) = 4.47, *p* = 0.035, *OR* = 0.71.

In contrast to Experiment 2, the separate logit analyses for D and U found no support for an interaction of instrumentality and personal force on either U, χ(1) = 2.00, *p* = 0.158, or on D, χ(1) = 1.98, *p* = 0.159.

Once again, we tested for sex differences in an extended mixed-effects analysis, but again we found no significant effects for sex, χ(1) = 0.83, *p* = 0.363, nor for the interaction of sex and parameter type, χ(1) = 2.85, *p* = 0.091. As in Experiment 2, both the D and U parameters were very similar for females and males (D: *M*_female_ = 0.59, *M*_male_ = 0.52; U: *M*_female_ = 0.36, *M*_male_ = 0.37).

As in Experiments 1 and 2, we also analyzed the conventional measure of moral inclinations, the relative frequency of utilitarian judgments. Consistent with the findings of Experiment 2, the approval of the harmful action was highest in the impersonal/side-effect condition and lowest in the personal/means condition (*M*s = 0.78 vs. 0.41, see also Table 2). A logit model with instrumentality and personal force as the independent variables revealed main effects of instrumentality, χ(1) = 81.02, *p* < 0.001, *OR* = 0.37, and personal force, χ(1) = 26.02, *p* < 0.001, *OR* = 0.56, but no significant interaction, χ(1) = 0.02, *p* = 0.885.

The finding of Experiments 1 and 2 of a moderate negative overall correlation between the PD parameters (H4a) was also replicated, *r* = −0.34, *p* < 0.001. Pairwise comparisons of the parameter correlations obtained in the four experimental conditions revealed that the correlation in the personal/means condition differed significantly from that in the impersonal/side-effect condition, *z* = 1.94, *p* = 0.026, and marginally significantly from the correlations in the other two conditions, personal/side-effect and impersonal/means, *z* = 1.43, *p* = 0.076, and *z* = 1.45, *p* = 0.073, respectively. The remaining three comparisons were not significant (all *z*s ≤ 0.52, all *p*s ≥ 0.301). Hence, with the larger sample size afforded by Experiment 3 (*N* = 75 per condition), hypothesis H4b (that parameter correlations differ between the experimental conditions) received statistical support.

### Discussion of Experiments 2 and 3

Experiments 2 and 3 replicated the main finding of the first study, a significant and large effect of the instrumentality manipulation on both deontological (H1a) and utilitarian inclinations (H1b). Again, instrumentality affected both utilitarian and deontological inclinations in opposite ways. While this finding is inconsistent with our predictions (derived from the modular myopia hypothesis), it is consistent with a “soft” version of the dual-process model (Skitka and Conway, 2019), according to which affective processes can influence both inclinations.

In line with H2, the D parameter was greater in personal than impersonal dilemmas, whereas the U parameter was greater in impersonal than personal dilemmas.

H3 predicted an interaction between instrumentality and personal force on one or both PD parameters. In line with this hypothesis, a significant interaction effect was obtained for U in Experiment 2; however, this interaction failed to replicate in Experiment 3. Overall, therefore, there was no convincing support for H3.

Interestingly, Experiment 3 also failed to replicate the significant interaction between instrumentality and personal force for the conventional measure of moral inclinations observed in Experiment 2. Given the much larger sample size and the associated higher power of Experiment 3, an interaction effect of the same size as in Experiment 2 should have shown up if it exists. The fact that we did not find it therefore suggests that the interaction found in Experiment 2 was a false positive. However, the possibility remains that the failure to obtain the interaction in Experiment 3 was due to factors associated with the experimental setting, the different scenarios used, or the different dependent variable (own likely action rather than a third-person moral evaluation).

Finally, Experiments 2 and 3 provided further support for the assumption that deontological and utilitarian inclinations are relatively independent of each other (Conway and Gawronski, 2013; Friesdorf et al., 2015). This support consists of three findings of our studies. First, the instrumentality of the harm and personal force influenced both deontological and utilitarian inclinations, but in opposite directions and to different degrees. Second, the two PD parameters were overall (i.e., across the experimental conditions) only moderately correlated. If the two underlying processes were reciprocally related, as implicitly assumed by the conventional measure of moral inclinations, this correlation should be much higher. Third, the correlation between the two PD parameters was found to be significantly different between at least two experimental conditions in Experiment 3. In Experiments 1 and 2, differences of similar size were obtained, suggesting that the lack of the between-condition differences in these studies was likely due to the comparatively small sample size.

In sum, the findings of Experiments 2 and 3 were largely consistent with each other, and their results also agreed for the most part with findings of Experiment 1. This enhanced confidence in the obtained results and the main conclusions drawn from them.

## General Discussion

Three experiments with a combined sample size of *N* = 449 were conducted to investigate the effects of two moral dilemma conceptualization variables, the instrumentality of the harm (means vs. side-effect) and personal force (personal vs. impersonal), on deontological and utilitarian inclinations in harm-related moral dilemmas. The process dissociation procedure (PD) was used to accommodate recent criticisms of the conventionally used, one-dimensional measure of moral inclinations (Conway and Gawronski, 2013). In agreement with previous studies reviewed in the introduction, the PD procedure proved to be a more sensitive measurement instrument than the conventional measure for detecting the contributions of deontological and utilitarian inclinations to moral judgment. Specifically, the PD procedure, but not the conventional measure of moral inclinations, revealed selective effects of the manipulated dilemma conceptualization variables on deontological and utilitarian inclinations. These findings, as well as the low obtained correlations between the two PD parameters, provide further support for the assumption that these judgment inclinations (and presumably, the underlying mental processes) are largely independent, a central claim of the dual-process model of moral judgment (Greene, 2007; Conway et al., 2018a).

A summary of the hypotheses tested and the results obtained in the present research is contained in Table 1.

The central question of our studies was to clarify whether instrumentality and personal force, two of the most important variables that influence judgments and choices in moral dilemma scenarios, exert their effects by influencing deontological inclinations, utilitarian inclinations, or both. This question can now be answered as follows: Instrumentality and personal force influence both inclinations, but in opposite directions and to different degrees. Specifically, personal dilemmas and dilemmas characterized by harm as a means evoked higher deontological tendencies and lower utilitarian tendencies than impersonal dilemmas and dilemmas where the harm was a side-effect. No convincing evidence was obtained for hypothesis H3, which predicted an interaction effect of instrumentality and personal force on one or both PD parameters.

In line with hypothesis H4b, Experiment 3 found that the parameter correlations differed significantly between at least some experimental conditions if a sufficient sample size is used. This finding seems at first sight to be at odds with the meta-analysis of Friesdorf et al. (2015) of the PD parameter correlations obtained in 40 studies. Similar to our findings, Friesdorf et al. (2015) found that the parameter correlations varied considerably (from *r* = −0.31 to *r* = 0.36), but they were unable to identify moderators that accounted for this variation. In contrast, our findings suggest that the parameter correlations depend to some degree on the combination of instrumentality and personal force. This factor may have remained undetected in the meta-analysis of Friesdorf et al. (2015) because most of the included studies used the same set of dilemmas (that introduced by Conway and Gawronski, 2013), which includes moral dilemmas from different content domains; furthermore, the included harm-related scenarios do not systematically differ with regard to the levels of instrumentality and personal force. Our studies refine the conclusions of Friesdorf et al. (2015) by suggesting that deontological and utilitarian inclinations are uncorrelated in impersonal situations where harm is a side-effect, but moderately negatively correlated in personal dilemmas where harm is a means. A possible explanation of this finding is that the latter scenarios were experienced as particularly conflictive and therefore motivated attempts to justify the chosen action (predominantly, harm-rejection) by devaluating the alternative action (outcome-maximization), with the degree of devaluation being proportional to the strength of the harm-rejection tendency. This devaluation process then manifested itself as a negative correlation of the PD parameters.

In our experiments, the PD parameters were derived from the participants’ answers to the question whether they would perform the harm-causing action themselves. That is, like several previous moral dilemma studies (e.g., Bartels, 2008; Christensen et al., 2014), our experiments focused on moral choice. In contrast, in probably the majority of previous moral dilemma studies, the participants were asked to judge the moral appropriateness of the harmful action, either from a first-person perspective (e.g., Conway and Gawronski, 2013), or from a third-person perspective (e.g., Greene et al., 2009). Can our findings, obtained for first-person choices, be generalized to moral evaluations? A positive answer is suggested by the consideration that, even though moral evaluation and choice are in part influenced by distinct factors, they are ultimately based on the same kinds of processes (i.e., deontological and utilitarian inclinations). For evaluations made from the first-person perspective at least, this assumption is supported by our finding that the harm approval rates obtained in our experiments were very similar to those obtained in previous PD studies asking for judgments of appropriateness (e.g., Conway and Gawronski, 2013). Additional empirical support for the assumption that choice and evaluation are closely related comes from a study by Bostyn et al. (2018), who found that choices in hypothetical moral dilemmas (participants decided whether or not to divert electroshocks from a cage with five mice to a single mouse) were predicted by moral appropriateness judgments as measured by a traditional moral dilemma battery. However, other studies (e.g., Tassy et al., 2013) found that people are more approving of harmful actions if they need to decide what to do than if they judge the action’s appropriateness from a third-person perspective. It is therefore well posssible that lower approval rates would have been found in our studies if people had been asked to make moral evaluations. However, this does not necessarily mean that manipulations of instrumentality and personal force influence moral choice and third-person moral evaluations in different ways. To answer this question, future studies could directly compare the two questions for the scenarios used in our studies, while also varying the degree of context information (see FeldmanHall et al., 2012).

In agreement with several previous studies (Friesdorf et al., 2015; Conway et al., 2018a; Armstrong et al., 2019), Experiment 1 found that the D parameter was higher for females than males; however, this finding was not replicated in Experiments 2 and 3, where personal force was included as a second factor. This difference to previous findings may have been due to the different set of moral dilemmas used. Alternatively, or in addition, it could reflect cultural differences (see, e.g., Graham et al., 2016) between the US samples of prior research and our German samples. So far, cultural variations of deontological and utilitarian inclinations have to our knowledge not been systematically studied. It is noteworthy, however, that in a related research area on the so-called *side-effect effect* (e.g., Knobe, 2003), Lau and Reisenzein (2016) found only a weak and strongly scenario-dependent effect for German samples.

The PD analysis of the effects of instrumentality provided a test of the modular myopia hypothesis, proposed by Greene (2014) to explain why a harmful action in a moral dilemma is more likely to be approved of if the harm is an unintended side-effect than if it is a means to achieve the goal of preventing greater harm. Based on this hypothesis, we predicted that a manipulation of the instrumentality of the harm would influence the D parameter but not the U parameter. In conflict with this prediction, the manipulation of instrumentality was found to affect both deontological and utilitarian inclinations. It should be noted, however, that our test of the modular myopia hypothesis has some limitations. Among others, the moral dilemmas used in our studies varied the personal force factor within two distinct sets of means and side-effect dilemmas. Varying both instrumentality and personal force within the same moral dilemma (Greene et al., 2009) would provide a stronger test of the modular myopia hypothesis.

Although instrumentality and personal force are among the most important determinants of moral judgments and choices (e.g., Christensen et al., 2014; Feltz and May, 2017), several additional dilemma conceptualization variables have been identified. For instance, Rosas and Koenigs (2014) found that self-interest (i.e., whether the agent in a moral dilemma is the victim or beneficiary of the harm-causing action) is another important feature of moral dilemmas that influences judgment and choice (see also Christensen et al., 2014). The dilemmas used in our studies were (roughly) counterbalanced within conditions with regard to self-interest, and thus the effects of this variable were controlled. However, in future PD studies, self-interest should be systematically varied to test whether it interacts with instrumentality and personal force. The same should be done for other important dilemma conceptualization variables, that have so far not been investigated with the PD procedure, such as the (in-) evitability of the harm resulting from the proposed action (see e.g., Christensen and Gomila, 2012). Finally, our research did not consider individual differences that affect judgments and actions in moral dilemmas, such as the tendency to prefer inaction over action. For a recent suggestion how the general preference for inaction and other individual difference variables can be taken into account, see Gawronski et al. (2017), and Körner et al. (2020).

## Conclusion

Three experiments used the process dissociation procedure to examine the effects of the instrumentality of the harm (means vs. side-effect) and personal force (personal vs. impersonal) on deontological and utilitarian inclinations in harm-related moral dilemmas. The results indicated that instrumentality and personal force affect both deontological and utilitarian inclinations, but in opposite directions and to different degrees. Taken together, our findings are consistent with a “soft” version of the dual-process theory of moral judgments, according to which affective and deliberative processes affect both deontological and utilitarian inclinations.

## Data Availability Statement

The raw data of the experiments reported in this article will be made available by the authors, without undue reservations, to any qualified researcher.

## Ethics Statement

All experiments were designed and conducted in accordance with the Code of Ethics of the World Medical Association (Declaration of Helsinki, British Medical Journal, July 18, 1964). All participants in all three experiments provided their written informed consent to participate. Ethical review and approval was not required for these studies in accordance with the German legislation and with institutional requirements. In the description of our experiments, we report all measures, manipulations, and exclusions (if any).

## Author Contributions

JL developed the study design and experimental materials. AH and RR helped to refine the design and materials. JL collected the data, performed the analyses, and wrote the first draft of the manuscript. JL, AH, and RR edited the final manuscript.

## Conflict of Interest

The authors declare that the research was conducted in the absence of any commercial or financial relationships that could be construed as a potential conflict of interest.

## Footnotes

1 We thank a reviewer for pointing out that logistic regression is better suited to our data.

2 The *z*-transformation used by Conway and Gawronski (2013), being a linear transformation, is unsuited to deal with the statistical peculiarities of probabilities as dependent variables. Actually, Conway and Gawronski (2013, p. 235) recommended the z-transformation for a different purpose, to make the two PD parameters better comparable to each other, arguing that in their raw form, they have different metrics, with the PD Utilitarianism parameter ranging between –1 and 1 and the PD Deontology parameter between 0 and 1. In fact, however, both parameters can *validly* vary only between 0 and 1, as both are probabilities, and the equations used to derive them from the data constrain both parameters to [0, 1] if the relative judgment frequencies are error-free. The *z*-transformation is therefore unnecessary. The untransformed parameters have the advantage that they have a clear meaning (they are probabilities) and an absolute scale level. These advantages are preserved in the logit analysis.

3 These exclusions were not pre-registered but are nevertheless justified to improve the data quality. We repeated all analyses with the complete sample (i.e., without the exclusions) and found only minute differences to the results reported here.
