# Supplementary material for: Effects of Instrumentality and Personal Force on Deontological and Utilitarian Inclinations in Harm-Related Moral Dilemmas
Source: Front Psychol. 2020 Jun 19;11:1222. doi: 10.3389/fpsyg.2020.01222 (PMC7318801; doi:10.3389/fpsyg.2020.01222)
Supplement: Supplementary file 1 [file Data_Sheet_1.PDF]

Online Supplementary Material: Moral Dilemmas

Ludwig, J., Reisenzein, R., & Hiemisch, A. *Effects of instrumentality and personal force on deontological and utilitarian inclinations in harm-related moral dilemmas.*

The following pages contain the moral dilemmas used in Experiments 1-3. The dilemmas were compiled from three sources (Christensen, Flexas, Calabrese, Gut, & Gomila, 2014; Conway & Gawronski, 2013; Cushman, Young, & Hauser, 2006) and translated to German. The English dilemmas presented below are re-translated from the adapted German versions and may thus differ slightly from the wording in the original source. The German language dilemmas are available from Jonas Ludwig, mail[at]jonasludwig.de, upon request.

The dilemmas vary along the dimensions of instrumentality (harm as a means versus a side-effect) and personal force (personal versus impersonal). Table 1A contains an overview of the 40 dilemmas, their levels of instrumentality and personal force, the source, and in which experiments of the present research they were deployed.

Table 2A contains the full text of the incongruent and congruent versions of the 40 dilemmas.

References

Christensen, J. F., Flexas, A., Calabrese, M., Gut, N. K., & Gomila, A. (2014). Moral judgment reloaded: A moral dilemma validation study. *Frontiers in Psychology*, *5*, 18. https://doi.org/10.3389/fpsyg.2014.00607

Conway, P., & Gawronski, B. (2013). Deontological and utilitarian inclinations in moral decision making: A process dissociation approach. *Journal of Personality and Social Psychology*, *104*(2), 216–235. https://doi.org/10.1037/a0031021

Cushman, F., Young, L., & Hauser, M. (2006). The role of conscious reasoning and intuition in moral judgment: Testing three principles of harm. *Psychological Science*, *17*(12), 1082–1089. https://doi.org/10.1111/j.1467-9280.2006.01834.x

Table 1A. Overview of the dilemmas and their levels of instrumantilty and personal force.

ID	Title	Instrumentality	Personal Force	Source (base version)	Exp.
CH01	Crying Baby	Side-effect	Personal	Christensen et al., 2014	1, 2, 3
CH02	Crying Baby	Side-effect	Impersonal	Christensen et al., 2014	2, 3
CH03	Submarine	Side-effect	Personal	Christensen et al., 2014	1, 2, 3
CH04	Submarine	Side-effect	Impersonal	Christensen et al., 2014	2, 3
CH05	Orphanage	Means	Personal	Christensen et al., 2014	1, 2, 3
CH06	Orphanage	Means	Impersonal	Christensen et al., 2014	2, 3
CH07	Ebola	Means	Personal	Christensen et al., 2014	1, 2, 3
CH08	Ebola	Means	Impersonal	Christensen et al., 2014	2, 3
CH09	Helicopter	Means	Personal	Christensen et al., 2014	1, 2, 3
CH10	Helicopter	Means	Impersonal	Christensen et al., 2014	2, 3
CH11	Space	Side-effect	Personal	Christensen et al., 2014	1, 2, 3
CH12	Space	Side-effect	Impersonal	Christensen et al., 2014	2, 3
CH13	Bus Driver	Side-effect	Personal	Christensen et al., 2014	1, 2, 3
CH14	Bus Driver	Side-effect	Impersonal	Christensen et al., 2014	2, 3
CH15	Container	Side-effect	Personal	Christensen et al., 2014	1, 2, 3
CH16	Container	Side-effect	Impersonal	Christensen et al., 2014	2, 3
CH17	Epidemic	Side-effect	Personal	Christensen et al., 2014	1, 2, 3
CH18	Epidemic	Side-effect	Impersonal	Christensen et al., 2014	2, 3
CH19	Left Behind	Means	Personal	Christensen et al., 2014	1, 2, 3
CH20	Left Behind	Means	Impersonal	Christensen et al., 2014	2, 3
CH21	Mine Worker	Means	Personal	Christensen et al., 2014	1, 2, 3
CH22	Mine Worker	Means	Impersonal	Christensen et al., 2014	2, 3
CH23	Rich Man	Means	Personal	Christensen et al., 2014	1, 2, 3
CH24	Rich Man	Means	Impersonal	Christensen et al., 2014	2, 3
CH25	Spy	Means	Personal	Christensen et al., 2014	1, 2, 3
CH26	Spy	Means	Impersonal	Christensen et al., 2014	2, 3
CU01	Aquarium	Side-effect	Personal	Cushman et al., 2006	1, 2, 3
CU02	Aquarium	Side-effect	Impersonal	Cushman et al., 2006	2, 3
CU03	Boxcar	Means	Personal	Cushman et al., 2006	1, 2, 3
CU04	Boxcar	Means	Impersonal	Cushman et al., 2006	2, 3
CU05	Burning House	Side-effect	Personal	Cushman et al., 2006	1, 2, 3
CU06	Burning House	Side-effect	Impersonal	Cushman et al., 2006	2, 3
CU07	Burning House	Means	Personal	Cushman et al., 2006	1, 2, 3
CU08	Burning House	Means	Impersonal	Cushman et al., 2006	2, 3
CU09	Rubble	Side-effect	Personal	Cushman et al., 2006	1, 2, 3
CU10	Rubble	Side-effect	Impersonal	Cushman et al., 2006	2, 3
CU11	Speedboat	Means	Personal	Cushman et al., 2006	1, 2, 3
CU12	Speedboat	Means	Impersonal	Cushman et al., 2006	2, 3
CO01	Car Accident	Side-effect	Personal	Conway & Gawronski, 2013	1, 2, 3
CO02	Car Accident	Side-effect	Impersonal	Conway & Gawronski, 2013	2, 3

Table 2A. Incongruent and congruent versions of 40 moral dilemmas.

ID	Incongruent Version	Congruent Version
**CH01**	Enemy soldiers have occupied your town and threaten to kill all civilians. You, your baby, and ten neighbors are hiding in the basement of a large house. Suddenly, you hear the voices of soldiers who have entered the house in search of valuables to loot. Instantly, your baby begins to cry loudly. The crying will attract the attention of the soldiers and they will kill you and the other members of your group.<br><br>If you hold the baby's mouth shut its screams will be muffled but the baby will suffocate. This will kill the baby but save you and your ten neighbors.<br><br>Do you hold the baby's mouth shut, causing it to suffocate in order to save yourself and your ten neighbors from the enemy soldiers?	Enemy soldiers have occupied your town and will deport all civilians to a labor camp in a nearby mine. You, your baby, and ten neighbors are hiding in the basement of a large house. Suddenly, you hear the voices of soldiers who have entered the house in search of valuables to loot. Instantly, your baby begins to cry loudly. The crying will attract the attention of the soldiers and they will capture you and the other members of your group.<br><br>If you hold the baby's mouth shut its screams will be muffled but the baby will suffocate. This will kill the baby but it will spare you and your ten neighbors from the labor camp.<br><br>Do you hold the baby's mouth shut, causing it to suffocate, to prevent the soldiers from finding you, your baby and the ten neighbors?
**CH02**	Enemy soldiers have occupied your town and threaten to kill all civilians. You, your baby, and ten neighbors are hiding in the basement of a large house. Suddenly, you hear the voices of soldiers who have entered the house to search for valuables to loot. In this instance your baby begins to cry loudly. The crying will attract the attention of the soldiers and they will kill you and the other members of your group.<br><br>If you turn on a loud furnace, the baby's screams will be muffled. However, the furnace creates a level of heat that is extremely unpleasant for children and adults but deadly for babies. This will kill your baby but save you and your ten neighbors.<br><br>Do you turn on the furnace, creating extreme heat in order to prevent the soldiers from discovering you, your baby and the ten neighbors?	Enemy soldiers have occupied your town and will deport all civilians to a labor camp in a nearby mine. You, your baby, and ten neighbors are hiding in the basement of a large house. Suddenly, you hear the voices of soldiers who have entered the house in search of valuables to loot. Instantly, your baby begins to cry loudly. The crying will attract the attention of the soldiers and they will capture you and the other members of your group.<br><br>If you turn on a loud furnace, the baby's screams will be muffled. However, the furnace creates a level of heat that is extremely unpleasant for children and adults but deadly for babies. This will kill your baby but save you and your ten neighbors from the labor camp.<br><br>Do you turn on the furnace, creating extreme heat, in order to prevent the soldiers from discovering you, your baby and the ten neighbors?
**CH03**	You are a crew member on a submarine which is currently on a dive below a massive iceberg. An explosion in the engine room has damaged the submarine. Several colleagues are injured and the only passageway between the upper and lower level of the boat is buried beneath debris. You and three other survivors are currently on the upper level. However, there is insufficient oxygen for all of you to survive until the submarine reaches the surface of the water. Two colleagues are lying on the lower level, unconscious. There is sufficient oxygen in that part of the boat.	You are a crew member on a submarine which is currently on a dive below a large/massive iceberg. An explosion in the engine room has damaged the submarine. Several colleagues are injured and the only passageway between the upper and lower level of the boat is buried beneath debris. You and another survivor are currently on the upper level. However, there is insufficient oxygen for both of you to survive until the submarine reaches the surface of the water. Four colleagues are lying on the lower level, unconscious. There is sufficient oxygen in that part of the boat.

	You can open the emergency hatch connecting the two levels. This will allow air to stream in and you will have enough oxygen to reach the surface of the water. However, the hatch door is too heavy to securely hold. It will fall on your two colleagues on the lower level and kill them. You and the three survivors will be saved though.<br><br>Do you open the emergency hatch and allow it to fall on your two unconscious colleagues on the lower level in order to provide sufficient oxygen to yourself and the three other survivors?	You can open the emergency hatch connecting the two levels. This will allow air to stream in and you will have enough oxygen to reach the surface of the water. However, the hatch door is too heavy to securely hold. It will fall on your four colleagues on the lower level and kill them. You and the other survivor will be saved though.<br><br>Do you open the emergency hatch and allow it to fall on your two unconscious colleagues on the lower level in order to provide sufficient oxygen to yourself and the other survivor?
**CH04**	You are a crew member on a submarine which is currently on a dive below a massive iceberg. An explosion in the engine room has damaged the submarine. Several colleagues are injured and the only passageway between the upper and lower level of the boat is buried beneath debris. You and three other survivors are currently on the upper level. However, there is insufficient oxygen for three people to survive until the submarine reaches the surface of the water. Two colleagues are lying on the lower level unconscious. There is sufficient oxygen in that part of the boat.<br><br>You can operate an emergency switch to open an emergency hatch between the two levels. This will allow air to stream in and you will have sufficient oxygen to reach the surface of the water. However, the hatch door is too heavy to hold. It will fall on the two colleagues on the lower level and kill them but it will save you and the three other survivors.<br><br>Do you operate the emergency switch to open the emergency hatch door? The door will fall on the unconscious colleagues on the lower level in order for you and the other three survivors to get sufficient oxygen.	You are a crew member on a submarine which is currently on a dive below a massive iceberg. An explosion in the engine room has damaged the submarine. Several colleagues are injured and the only passageway between the upper and lower level of the boat is buried beneath debris. You and two other survivors are currently on the upper level. However, there is insufficient oxygen for three people to survive until the submarine reaches the surface of the water. Four colleagues are lying on the lower level, unconscious. There is sufficient oxygen in that part of the boat.<br><br>You can operate an emergency switch to open an emergency hatch between the two levels. This will allow air to stream in and you will have sufficient oxygen to reach the surface of the water. However, the hatch door is too heavy to hold. It will fall on the four colleagues on the lower level and kill them but it will save you and the other two survivors.<br><br>Do you operate the emergency switch to open the emergency hatch door? The door will fall on the four unconscious colleagues on the lower level in order for you and the other two survivors to get sufficient oxygen.
**CH05**	You work in an orphanage in a wartorn country. Rebels have overthrown the government and are killing everyone who does not support their regime. They are also committing a genocide against a specific ethnic group. You and eleven orphans living in the orphanage have not supported the rebellion. Furthermore, a child that is member of this ethnic group is living in the orphanage. There is no way for you and the orphans to escape. The soldiers have surrounded the orphanage and will begin their attack at daybreak.<br><br>If you suffocate the child that belongs to the persecuted ethnic group in its sleep, the soldiers will believe that you support their regime. This will kill the child but it will save you, the nurses of the orphanage and the other ten children.	You work in an orphanage in a wartorn country. Rebels have overthrown the government and are killing everyone who does not support their regime. They are also committing a genocide against a specific ethnic group. You and eleven orphans living in the orphanage have not supported the rebellion. Furthermore, eight children of this ethnic group are living in the orphanage. There is no way for you and the orphans to escape. The soldiers have surrounded the orphanage and will begin their attack at daybreak.<br><br>If you suffocate the children that belong to the persecuted ethnic group in their sleep, the soldiers will believe that you support their regime. This will kill the children but it will save you, the nurses of the orphanage and the other three children.

	Do you lead the soldiers to believe that you support their regime by suffocating the child in order for you, the nurses and the other ten children to survive?	Do you lead the soldiers to believe that you support their regime by suffocating the children in order for you, the nurses and the other three children to survive?
**CH06**	You work in an orphanage in a wartorn country. Rebels have overthrown the government and are killing everyone who does not support their regime. They are also committing a genocide against a specific ethnic group. You and eleven orphans living in the orphanage have not supported the rebellion. Furthermore, a child that is member of this ethnic group is living in the orphanage. There is no way for you and the orphans to escape. The soldiers have surrounded the orphanage and will begin their attack at daybreak.<br><br>If you order a nurse to administer an overdose of a fast-acting sleeping aid to the child of the persecuted ethnic minority, the soldiers will believe that you support their regime. This will kill the child but it will save you, the nurses and the other ten children.<br><br>Do you lead the soldiers to believe that support their regime by ordering the nurse to administer an overdose to the child in order to spare you, the nurses and the ten other children?	You work in an orphanage in a wartorn country. Rebels have overthrown the government and are killing everyone who does not support their regime. They are also committing a genocide against a specific ethnic group. You and eleven orphans living in the orphanage have not supported the rebellion. Furthermore, eight children that are members of the persecuted ethnic group are living in the orphanage. There is no way for you and the orphans to escape. The soldiers have surrounded the orphanage and will begin their attack at daybreak.<br><br>If you order a nurse to administer an overdose of a fast-acting sleeping aid to the children of the persecuted ethnic minority, the soldiers will believe that you support their regime. This will kill the children but it will save you, the nurses and the other three children.<br><br>Do you lead the soldiers to believe that support their regime by ordering the nurse to administer an overdose to the child in order to spare you, the nurses and the three other children?
**CH07**	You are a volunteer worker at a health center in an African village. A man from a neighboring village has contracted the Ebola virus. The illness caused by this virus is extremely contagious, incurable and almost always deadly. Unexpectedly, he has already survived for a month which probably means that he is immune to the virus and doesn't need any further treatment. However, he erroneously believes that he requires treatment at the health center and that he can be assisted there. You see him approaching and know: if he enters the village he will most likely infect the 119 inhabitants of the village. This will cause the vast majority of the inhabitants, unlike himself, to die of the illness.<br><br>If you shoot him with the gun belonging to the center, you will prevent him from infecting you and the rest of the village with the virus. This will kill him but it will save you and the 119 inhabitants of the village.<br><br>Do you prevent the man from entering the village by shooting him in order to save you and the other 119 inhabitants of the village from being infected?	You are a volunteer worker at a health center in an African village. A man from a neighboring village has contracted an Influenza virus. The illness caused by this virus is extremely contagious, but curable and only rarely deadly. He has already survived for a month which probably means that he is immune to the virus and doesn't need any further treatment. However, he erroneously believes that he requires treatment at the health center and that he can be assisted there. You see him approaching and know: if he enters the village he will most likely infect the 119 inhabitants of the village.<br><br>If you shoot him with the gun belonging to the center, you will prevent him from infecting you and the rest of the village with the virus. This will kill him but it will prevent you and the 119 inhabitants of the village from contracting the virus.<br><br>Do you prevent the man from entering the village by shooting him in order to save you and the other 119 inhabitants of the village from being infected?
**CH08**	You are a volunteer worker at a health center in an African village. A man from a neighboring village has contracted the Ebola virus. The illness caused by this virus is extremely contagious, incurable and almost always deadly. Unexpectedly, he has already survived for a month which probably means that he is immune tot he virus and doesn't need any further treatment. However, he erroneously	You are a volunteer worker at a health center in an African village. A man from a neighboring village has contracted an Influenza virus. The illness caused by this virus is extremely contagious, but curable and only rarely deadly. He has already survived for a month which probably means that he is immune to the virus and doesn't need any further treatment. However, he erroneously believes that he

	believes that he requires treatment at the health center and that he can be assisted there. You see him approaching and know: if he enters the village he will most likely infect the 119 inhabitants of the village. This will cause the vast majority of the inhabitants, unlike himself, to die of the illness.<br><br>You can however inform the municipal council about the problem. The council will certainly then order the shooting of the approaching infected person to prevent him from infecting you and the rest of the village with the virus. This will kill him but it will save you and the 119 inhabitants of the village.<br><br>Do you inform the municipal council so it can order the shooting of the man in order to prevent him from infecting you and the 119 inhabitants?	requires treatment at the health center and that he can be assisted there. You see him approaching and know: if he enters the village he will most likely infect the 119 inhabitants of the village.<br><br>You can however inform the municipal council about the problem. The council will certainly then order the shooting of the approaching infected person to prevent him from infecting you and the rest of the village with the virus. This will kill him but it will prevent you and the 119 inhabitants of the village from contracting the virus.<br><br>Do you inform the municipal council so it can order the shooting of the man in order to prevent him from infecting you and the 119 inhabitants?
**CH09**	You are a doctor aboard a rescue helicopter on duty during a devastating flood disaster. You have saved nine severely injured people and are working to keep them alive when the pilot announces an engine failure. He orders the helicopter to be rid of ballast to prevent it from crashing and killing everyone on board. You cannot leave the helicopter because the injured people need your assistance and the heavy medical equipment is securely screwed down.<br><br>You can push an injured person overboard in order to reduce the weight and keep the helicopter from crashing. This will kill the injured person but it will save you, the pilot and the other eight injured people.<br><br>Do you push the injured person overboard to reduce weight and keep the helicopter with you, the pilot and the eight other injured people aboard from crashing?	You are a doctor aboard a rescue helicopter on duty during a devastating flood disaster. You have saved three severely injured people and are working to keep them alive when the pilot announces an engine failure. He orders the helicopter to be rid of ballast to prevent it from crashing and killing everyone on board. You cannot leave the helicopter because the injured people need your assistance and the heavy medical equipment is securely screwed down.<br><br>You can push the three injured people overboard in order to reduce the weight and keep the helicopter from crashing. This will kill the injured people but it will save you and the pilot.<br><br>Do you push the three injured people overboard to reduce weight and keep the helicopter with you and the pilot aboard from crashing?
**CH10**	You are a doctor aboard a rescue helicopter on duty during a devastating flood disaster. You have saved nine severely injured people and are working to keep them alive when the pilot announces an engine failure. He orders the helicopter to be rid of ballast to prevent it from crashing and killing everyone on board. You cannot leave the helicopter because the injured people need your assistance and the heavy medical equipment is securely screwed down.<br><br>You can also cut the rope that is currently being used to lift the last injured person up to the helicopter in order to reduce the weight and keep the helicopter from crashing. This will kill the injured person but it will save you, the pilot and the eight other injured people.	You are a doctor aboard a rescue helicopter on duty during a devastating flood disaster. You have saved three severely injured people and are working to keep them alive when the pilot announces an engine failure. He orders the helicopter to be rid of ballast to prevent it from crashing and killing everyone on board. You cannot leave the helicopter because the injured people need your assistance and the heavy medical equipment is securely screwed down.<br><br>You can also cut the rope that is currently being used to lift the three injured people up to the helicopter in order to reduce the weight and keep the helicopter from crashing. This will kill the injured people but it will save you and the pilot.<br><br>Do you cut the rope that is being used to lift the three people up to the helicopter in order to reduce the weight and keep the helicopter with you and the pilot from crashing?

	Do you cut the rope that is being used to lift the last person up to the helicopter in order to reduce the weight and keep the helicopter with you, the pilot and the eight other injured people aboard from crashing?	
**CH11**	You are working as an engineer on the International Space Station ISS when a fire suddenly breaks out in the cargo bay. The automatic security system can release oxygen from this area in order to put out the fire. However, this will open the exterior door and that only works if the inner hatch is closed. On the other side of the hatch remains a mechanic doing repair work. You realize that he does not have enough time to take off the heavy equipment that is attached to his suit. He is trying to squeeze through the inner hatchway with his equipment but will get stuck. This will cause the fire to rapidly spread and kill you and the ten other astronauts aboard the ISS.<br><br>You can shove the mechanic back into the cargo bay. This will allow you to close the inner hatch, activating the emergency system and extinguishing the fire. This will eject the mechanic into space and kill him but it will save you and the ten other astronauts.<br><br>Do you shove the mechanic back into the cargo bay in order to put out the fire? The emergency system will be activated and the mechanic will be ejected into space.	You are working as an engineer on the International Space Station ISS when a fire suddenly breaks out in the cargo bay. The automatic security system can release oxygen from this area in order to put out the fire. However, this will open the exterior door and that only works if the inner hatch is closed. On the other side of the hatch remains a mechanic doing repair work. You realize that he does not have enough time to take off the heavy equipment that is attached to his suit. He is trying to squeeze through the inner hatchway with his equipment but will get stuck. The fire will spread rapidly and cause great damage. The station will become temporarily uninhabitable and you will have to interrupt your mission.<br><br>You can shove the mechanic back into the cargo bay. This will allow you to close the inner hatch, activating the emergency system and extinguishing the fire. This will eject the mechanic into space and kill him but it will extinguish the fire and you will not have to interrupt your mission.<br><br>Do you shove the mechanic back into the cargo bay in order to put out the fire? The emergency system will be activated and the mechanic will be ejected into space.
**CH12**	You are working as an engineer on the International Space Station ISS when a fire suddenly breaks out in the cargo bay. The automatic security system can release oxygen from this area in order to put out the fire. However, this will open the exterior door and that only works if the inner hatch is closed. On the other side of the hatch remains a mechanic doing repair work. You realize that he does not have enough time to take off the heavy equipment that is attached to his suit. He is trying to squeeze through the inner hatchway with his equipment but will get stuck. This will cause the fire to rapidly spread and kill you and the ten other astronauts aboard the ISS.<br><br>You can operate a switch that closes the inner hatch before the mechanic reaches the hatchway. This will activate the emergency system and extinguish the fire. The mechanic will be ejected into space and killed but you and the ten other astronauts will be saved.<br><br>Do you operate the switch that closes the inner hatch in order to put out the fire? This will activate the emergency system causing the mechanic to be ejected into space.	You are working as an engineer on the International Space Station ISS when a fire suddenly breaks out in the cargo bay. The automatic security system can release oxygen from this area in order to put out the fire. However, this will open the exterior door and that only works if the inner hatch is closed. On the other side of the hatch remains a mechanic doing repair work. You realize that he does not have enough time to take off the heavy equipment that is attached to his suit. He is trying to squeeze through the inner hatchway with his equipment but will get stuck. The fire will spread rapidly and cause great damage. The station will become temporarily uninhabitable and you will have to interrupt your mission.<br><br>You can operate a switch that closes the inner hatch before the mechanic reaches the hatchway. This will activate the emergency system and extinguish the fire. The mechanic will be ejected into space and killed but you will not have to interrupt your mission.<br><br>Do you operate the switch that closes the inner hatch in order to put out the fire? This will activate the emergency system causing the mechanic to be ejected into space.

**CH13**	You are a bus driver on your way to the zoo with a group of kindergarten kids. Suddenly, your tire bursts and the bus plunges off a bridge into a river. You and three children are still aboard the sinking vehicle. The others have already been swept away. You pick up two kid and try to swim to the bus door. The third kid hangs on to your leg. You realize that you are not strong enough to fight against the current carrying all three children.<br><br>If you shake off the child that is hanging onto your leg you will be able to get to the bus door and save the two children. The third child, however, will be swept away and drown.<br><br>Do you shake off the third child so it lets go of your leg in order to save yourself and the other two children? This will cause the third child to be swept away by the current and killed.	You are a bus driver on your way to the zoo with a group of kindergarten kids. Suddenly, your tire bursts and the bus plunges off a bridge into a river. You and three children are still aboard the sinking vehicle. The others have already been swept away. You swim towards the bus door but notice that the three children are hanging onto your leg. You realize that you are not strong enough to fight against the current carrying all three children.<br><br>If you shake off the children that are hanging onto your leg you will be able to get to the bus door. You will be able to save yourself but the children will be swept away and drown.<br><br>Do you shake off the children so they let go of your leg, causing them to be swept away by the current, in order to save yourself?
**CH14**	You are a bus driver on your way to the zoo with a group of kindergarten kids. Suddenly, your tire bursts and the bus plunges off a bridge into a river. You and three children are still aboard the sinking vehicle. The others have already been swept away. You pick up two kid and try to swim to the bus door. The third kid hangs on to your leg. You realize that you are not strong enough to fight against the current carrying all three children.<br><br>If you move the seats out of the way, you can clear the path to the exit door. But you will also block the aisle and the third child will be trapped. The child will drown but you will be able to save yourself and the two other children.<br><br>Do you move the seats that are blocking the door out of the way in order to help yourself and the two other children reach the exit? This will trap the third child and it will drown.	You are a bus driver on your way to the zoo with a group of kindergarten kids. Suddenly, your tire bursts and the bus plunges off a bridge into a river. You and three children are still aboard the sinking vehicle. The others have already been swept away. You swim towards the bus door but notice that the exit is blocked by several destroyed seats.<br><br>If you move the seats out of the way, you can clear the path to the exit door. But you will also block the aisle and the three children will be trapped. The children will drown but you will be able to save yourself.<br><br>Do you move the seats that are blocking the exit out of the way in order to reach the door? This will trap the children.
**CH15**	You are part of a team of dock workers that is tasked with attaching cranes‘ chains to freighters so they can be unloaded. You and your colleagues are in the process of attaching chains to a container and are climbing on top to make sure it is being properly unloaded. As the crane begins lifting the container a red emergency light flashes, indicating that the chains will fail because the container is swaying too much. You see that this is being caused by two colleagues who are arguing atop the container. The container is swaying dangerously above five colleagues who are working on the deck below. If the chains fail as it hovers over the deck due to the heavy swaying, the container will fall on the five colleagues and kill them.	You are part of a team of dock workers that is tasked with attaching cranes‘ chains to freighters so they can be unloaded. You and your colleagues are in the process of attaching chains to a container and are climbing on top to make sure it is being properly unloaded. As the crane begins lifting the container a red emergency light flashes, indicating that the chains will fail because the container is swaying too much. You see that this is being caused by two colleagues who are arguing atop the container. The container is swaying dangerously above a colleague who is working on the deck below. If the chains fail as it hovers over the deck due to the heavy swaying, the container will fall on the colleague and kill him.

	If you push the two arguing colleagues off the container, you can prevent the container from swaying, its chains from failing and the container from falling. However, one of the arguing colleagues is not wearing his safety harness and will fall. This will kill him but it will save the five colleagues on the deck below.<br>Do you push the arguing colleagues off the container which will cause the one not wearing a safety harness to fall? This will stop the container from swaying and prevent it from falling on the five colleagues on the deck.	If you push the two arguing colleagues off the container, you can prevent the container from swaying, its chains from failing and the container from falling. However, neither of the arguing colleagues are wearing their safety harness and will fall off the container. Pushing them will kill them but it will save the colleague on the deck below.<br>Do you push the arguing colleagues off the container which will cause both of them to fall? This will stop the container from swaying and prevent it from falling on the colleague on the deck.
**CH16**	You are part of a team of dock workers that is tasked with attaching cranes‘ chains to freighters so they can be unloaded. You and your colleagues are in the process of attaching chains to a container and are climbing on top to make sure it is being properly unloaded. As the crane begins lifting the container a red emergency light flashes, indicating that the chains will fail because the container is swaying too much. You see that this is being caused by two colleagues who are arguing atop the container. The container is swaying dangerously above five colleagues who are working on the deck below. If the chains fail as it hovers over the deck due to the heavy swaying, the container will fall on the five colleagues and kill them.<br>If you operate the emergency switch, the container will be dropped back into the cargo area. You and your colleagues on the container are secured by safety harnesses. However, a worker is already unloading the next shipping container in the cargo bay beneath the container. The falling container will crush him but it will save the five colleagues on deck.<br>Do you operate the emergency switch, causing the container to fall back into the cargo area? The container will crush the colleague working there but the five colleagues on deck will be saved.	You are part of a team of dock workers that is tasked with attaching cranes‘ chains to freighters so they can be unloaded. You and your colleagues are in the process of attaching chains to a container and are climbing on top to make sure it is being properly unloaded. As the crane begins lifting the container a red emergency light flashes, indicating that the chains will fail because the container is swaying too much. The container is swaying dangerously above a colleague who is working on the deck below. If the chains fail as it hovers over the deck due to the heavy swaying, the container will fall on the colleague and kill him.<br>If you operate the emergency switch, the container will be dropped back into the cargo area. You and your colleagues on the container are secured by safety harnesses. However, five workers are already unloading the next shipping container in the cargo bay beneath the container. The falling container will crush them but it will save the colleague on deck.<br>Do you operate the emergency switch, causing the container to fall back into the cargo area? The container will crush the five workers in the cargo area but the colleague on deck will be saved.
**CH17**	An epidemic has spread across the entire planet and has killed thousands of people. The virus is extremely contagious, nearly incurable, causes severe health ailments and is almost always fatal. You are a researcher and have developed two substances in a laboratory. Unfortunately, both have mistakenly been labelled “vaccine“. One vial indeed contains the vaccine that could save thousands of lives, the other contains a deadly substance.<br>If you inject two lab assistants with a dose of each of the two substances, you will be able to identify the vaccine. One of the lab assistans will die but you will be able to save thousands of lives with the vaccine.	An epidemic has spread across the entire planet and has infected thousands of people. The virus is extremely contagious, but curable. It causes only minor health ailments and usually subsides without medical treatment after a few weeks. You are a researcher and have developed two substances in a laboratory. Unfortunately, both have mistakenly been labelled “vaccine“. One vial indeed contains the vaccine that could prevent thousands of new infections, the other contains a deadly substance.

	Do you inject your assistants with the substances in order to identify the vaccine and save thousands of lives? One vial contains the vaccine, the other contains a lethal substance.	If you inject two lab assistants with a dose of each of the two substances, you will be able to identify the vaccine. One of the lab assistans will die but you will be able to prevent thousands of new infections with the vaccine.<br><br>Do you inject your assistants with the substances in order to identify the vaccine and prevent thousands of new infections? One vial contains the vaccine, the other contains a lethal substance.
**CH18**	An epidemic has spread across the entire planet and has killed thousands of people. The virus is extremely contagious, nearly incurable, causes severe health ailments and is almost always fatal. You are a researcher and have developed two substances in a laboratory. Unfortunately, both have mistakenly been labelled “vaccine“. One vial indeed contains the vaccine that could save thousands of lives, the other contains a deadly substance.<br><br>If you have a nurse administer the injections to two patients, you will be able to identify the vaccine. One of the two patients will die but the vaccine will save thousands of lives.<br><br>Do you have the nurse administer the injections to two patients - one receiving the vaccine, the other a lethal substance - in order to identify the vaccine and vaccinate thousands of people?	An epidemic has spread across the entire planet and has infected thousands of people. The virus is extremely contagious, but curable. It causes only minor health ailments and usually subsides without medical treatment after a few weeks. You are a researcher and have developed two substances in a laboratory. Unfortunately, both have mistakenly been labelled “vaccine“. One vial indeed contains the vaccine that could prevent thousands of new infections, the other contains a deadly substance.<br><br>If you have a nurse administer the injections to two patients, you will be able to identify the vaccine. One of the two patients will die but the vaccine will prevent thousands of new infections.<br><br>Do you have the nurse administer the injections to two patients - one receiving the vaccine, the other a lethal substance - in order to identify the vaccine and vaccinate thousands of people?
**CH19**	You are the leader of a small group of soldiers. Returning from a mission in enemy territory, one of your men steps on a landmine and is severely injured. You cannot carry the injured soldier since this will slow you down significantly and you will risk being caught by the enemy soldiers. If you however decide to leave your comrade behind, enemy troops will capture and torture him until he reveals the location of your ally‘s important compound. Then your enemies will attack this compound and kill the ten ally soldiers that are stationed there. The soldier begs you not to leave him behind but the enemy troops are closing in on you and it is dangerous for you and your men to be held up any longer since you are all out of ammunition.<br><br>You can stab the injured soldier and prevent him from being captured and tortured, subsequently revealing information about your ally’s location. This will kill the soldier who has been injured by the landmine but it will save the ten ally troops.	You are the leader of a small group of soldiers. Returning from a mission in enemy territory, one of your men steps on a landmine. The explosion severely injures him and five fellow soldiers. You cannot carry the injured soldiers since this will slow you down significantly and you will risk being caught by the enemy soldiers. If you however decide to leave your comrades behind, enemy troops will capture and torture them until they reveal the location of your ally‘s important compound. Then your enemies will attack this compound and kill the three ally soldiers that are stationed there. The soldiers beg you not to leave them behind but the enemy troops are closing in on you and it is dangerous for you and your men to be held up any longer since you are all out of ammunition.<br><br>You can stab the injured soldiers and prevent them from being captured and tortured, subsequently revealing information about your ally’s location. This will kill the six soldiers who have been injured by the landmine but it will save the three ally troops.

	Do you prevent your injured comrade from being tortured by stabbing him yourself in order to keep him from revealing the location of your ally's ten troops?	Do you prevent your injured comrades from being tortured by stabbing them yourself in order to keep them from revealing the location of your ally's three troops?
**CH20**	You are the leader of a small group of soldiers. Returning from a mission in enemy territory, one of your men steps on a landmine and is severely injured. You cannot carry the injured soldier since this will slow you down significantly and you will risk being caught by the enemy soldiers. If you however decide to leave your comrade behind, enemy troops will capture and torture him until he reveals the location of your ally‘s important compound. Then your enemies will attack this compound and kill the ten ally soldiers that are stationed there. The soldier begs you not to leave him behind but the enemy troops are closing in on you and it is dangerous for you and your men to be held up any longer since you are all out of ammunition.<br><br>You can mark the position of the injured soldier with a flare rocket. This will cause the enemy to bomb the surrounding area and prevent your injured comrade from being tortured and revealing information about the location of your allies. It will kill the wounded soldier but it will save the ten allied troops.<br><br>Do you prevent the soldier who has been injured by the landmine from being tortured and revealing the location of the ten allied troops by making the enemy bomb the surrounding area?	You are the leader of a small group of soldiers. Returning from a mission in enemy territory, one of your men steps on a landmine. The explosion severely injures him and five fellow soldiers. You cannot carry the injured soldiers since this will slow you down significantly and you will risk being caught by the enemy soldiers. If you however decide to leave your comrades behind, enemy troops will capture and torture them until they reveal the location of your ally‘s important compound. Then your enemies will attack this compound and kill the three ally soldiers that are stationed there. The soldiers beg you not to leave them behind but the enemy troops are closing in on you and it is dangerous for you and your men to be held up any longer since you are all out of ammunition.<br><br>You can mark the position of the injured soldiers with a flare rocket. This will cause the enemy to bomb the surrounding area and prevent your injured comrades from being tortured and revealing information about the location of your allies. It will kill the six wounded soldiers but it will save the three allied troops.<br><br>Do you prevent the soldiers who have been injured by the landmine from being tortured and revealing the location of the three allied troops by making the enemy bomb the surrounding area?
**CH21**	You are a mineworker working in underground mining. The only way to reach the surface is by using a simple traction elevator that carries multiple cabins. Each cabin has the capacity to transport three people. Your workday has just ended and you are waiting to take a cabin to return above ground. Suddenly you notice that one of the cables that the cabins are attached to is about to rupture. All thirteen mineworkers currently aboard the various cabins are at risk of plummeting to death.<br><br>You can however detach the retainer of a cabin that is carrying a single mineworker. This would reduce the weight sufficiently in order to prevent the cable carrying the cabins of the twelve other mineworkers from rupturing. The mineworker in the cabin that you would cause to crash would die but you would save the twelve other mineworkers.<br><br>Do you detach the retainer of the cabin, causing the cabin carrying one worker to crash? This will reduce weight sufficiently to prevent the cable from rupturing and the cabins of the other twelve mineworkers from crashing.	You are a mineworker working in underground mining. The only way to reach the surface is by using a simple traction elevator that carries multiple cabins. Each cabin has the capacity to transport three people. Your workday has just ended and you are waiting to take a cabin to return above ground. Suddenly you notice that one of the cables that the cabins are attached to is about to rupture. All five mineworkers currently aboard the various cabins are at risk of plummeting to death.<br><br>You can however detach the retainer of a cabin that is carrying three mineworkers. This would reduce the weight sufficiently in order to prevent the cable carrying the cabins of the two other mineworkers from rupturing. The mineworkers in the cabin that you would cause to crash would die but you would save the two other mineworkers.<br><br>Do you detach the retainer of the cabin, causing the cabin carrying three workers to crash? This will reduce weight sufficiently to prevent the cable from rupturing and the cabin of the other two mineworkers from crashing.

**CH22**	You are a mineworker working in underground mining. The only way to reach the surface is by using a simple traction elevator that carries multiple cabins. Each cabin has the capacity to transport three people. Your workday has just ended and you are waiting to take a cabin to return above ground. Suddenly you notice that one of the cables that the cabins are attached to is about to rupture. All thirteen mineworkers currently aboard the various cabins are at risk of plummeting to death.<br><br>You can however operate an emergency switch that causes one cabin carrying a single mineworker to crash. This will reduce a sufficient amount of weight to prevent the cable carrying the cabins of the twelve other mineworkers from rupturing. The mineworker in the cabin that is crashed will die but you will save the twelve other mineworkers.<br><br>Do you operate the emergency switch, causing the cabin with the single worker to crash? This will reduce a sufficient amount of weight to prevent the cable from rupturing and the cabins with the twelve other mineworkers from crashing.	You are a mineworker working in underground mining. The only way to reach the surface is by using a simple traction elevator that carries multiple cabins. Each cabin has the capacity to transport three people. Your workday has just ended and you are waiting to take a cabin to return above ground. Suddenly you notice that one of the cables that the cabins are attached to is about to rupture. All five mineworkers currently aboard the various cabins are at risk of plummeting to death.<br><br>You can however operate an emergency switch that causes one cabin carrying three mineworkers to crash. This will reduce a sufficient amount of weight to prevent the cable carrying the cabins of the two other mineworkers from rupturing. The mineworkers in the cabin that is crashed will die but you will save the two other mineworkers.<br><br>Do you operate the emergency switch, causing the cabin with the three workers to crash? This will reduce a sufficient amount of weight to prevent the cable from rupturing and the cabin with the two other mineworkers from crashing.
**CH23**	You are an electrician working to repair a power line in the house of an industrial magnate. The man is suffering from an incurable form of cancer. It recently became public that he intends to donate his entire wealth to a local children‘s hospital after his death. This hospital is home to 109 sick children that could be saved with the money as they could be provided with a medication that would otherwise be prohibitively expensive. However, the children cannot wait for treatment much longer since they are terminally ill.<br><br>You can suffocate the industrial magnate in his sleep by covering his mouth and nose. This will accelerate the donation for the children. It will kill the magnate but it will save the lives of 109 children.<br><br>Do you cover the industrial magnate’s mouth and nose while he sleeps in order to accelerate the donation aiding the 109 sick children?	You are an electrician working to repair a power line in the house of an industrial magnate. The man is suffering from an incurable form of cancer. It recently became public that he intends to donate his entire wealth to a local children‘s hospital after his death. This hospital is home to 109 sick children. The money is intended to be used to construct a new playground in the inner courtyard.<br><br>You can suffocate the industrial magnate in his sleep by covering his mouth and nose. This will accelerate the donation for the children. It will kill the magnate but it will facilitate the construction of the playground for 109 children.<br><br>Do you cover the industrial magnate’s mouth and nose while he sleeps in order to accelerate the donation aiding the 109 sick children?
**CH24**	You are an electrician working to repair a power line in the house of an industrial magnate. The man is suffering from an incurable form of cancer. It recently became public that he intends to donate his entire wealth to a local children‘s hospital after his death. This hospital is home to 109 sick children that could be saved with the money as they could be provided with a medication that would otherwise be prohibitively expensive. However, the children cannot wait for treatment much longer since they are terminally ill.	You are an electrician working to repair a power line in the house of an industrial magnate. The man is suffering from an incurable form of cancer. It recently became public that he intends to donate his entire wealth to a local children‘s hospital after his death. This hospital is home to 109 sick children. The money is intended to be used to construct a new playground in the inner courtyard.<br><br>Instead of repairing the power line, you can short-circuit them, causing the magnate’s respirator to briefly malfunction. This will accelerate the donation to

	Instead of repairing the power line you can short-circuit them, causing the magnate's respirator to briefly malfunction. This will accelerate the donation to the children. It will kill the industrial magnate but it will save the lives of the 109 ill children.<br><br>Do you short-circuit the powerline and cause the magnate's respirator to malfunction in order to accelerate the donation intended to help the 109 ill children?	the children. It will kill the industrial magnate but it will facilitate the construction of the playground fort he 109 children.<br><br>Do you short-circuit the powerline and cause the magnate's respirator to malfunction in order to accelerate the donation intended to help the 109 ill children?
**CH25**	You are an army officer currently at war. Wedged under a collapsed building, your soldiers have discovered an important enemy spy. You have been informed via radio transmission that enemy soldiers are planning a surprise attack on a nearby city. Which city, however, you do not know. Thousands of civilians will die if you and your soldiers don't stop them. The spy knows which city is going to be targeted. You have interrogated him personally but he won't disclose the information. He will soon succumb to his injuries.<br><br>If you torture the spy he will disclose which city will be attacked. The torture will kill him but you will save thousands of civilians.<br><br>Do you torture the spy to make him reveal the information and stop the attack on thousands of civilians?	You are an army officer currently at war. Wedged under a collapsed building, your soldiers have discovered an important enemy spy. You have been informed via radio transmission that enemy soldiers in a nearby city are attempting to obtain classified information whose release would cause a political crisis for your government on an international level. However, you do not know in which city the enemy agents are stationed. The spy does have this knowledge. You have interrogated him personally but he won't disclose the information. He will soon succumb to his injuries.<br><br>If you torture the spy he will disclose the location of the enemy agents. The torture will kill him but you will provide a highly valuable service to your government.<br><br>Do you torture the spy to make him reveal the information and prevent your government from falling into political crisis?
**CH26**	You are an army officer currently at war. Wedged under a collapsed building, your soldiers have discovered an important enemy spy. You have been informed via radio transmission that enemy soldiers are planning a surprise attack on a nearby city. Which city, however, you do not know. Thousands of civilians will die if you and your soldiers don't stop them. The spy knows which city is going to be targeted. You have interrogated him personally but he won't disclose the information. He will soon succumb to his injuries. Rats are already gnawing on him and he is suffering tremendously.<br><br>If you let the rats continue, the spy will eventually disclose which city will be targeted. The torture will kill him but you will save thousands of civilians.<br><br>Do you let the rats continue to gnaw on the spy so he reveals his knowledge about the imminent attack? This will allow you to stop the attack on thousands of civilians.	You are an army officer currently at war. Wedged under a collapsed building, your soldiers have discovered an important enemy spy. You have been informed via radio transmission that enemy soldiers in a nearby city are attempting to obtain classified information whose release would cause a political crisis for your government on an international level. However, you do not know in which city the enemy agents are stationed. The spy does have this knowledge. You have interrogated him personally but he won't disclose the information. He will soon succumb to his injuries. Rats are already gnawing on him and he is suffering tremendously.<br><br>If you let the rats continue, the spy will eventually disclose the location of the enemy agents. The torture will kill him but you will provide a highly valuable service to your government.<br><br>Do you let the rats continue to gnaw on the spy so he reveals his knowledge in order to prevent your government from falling into political crisis?

**CU01**	You are at the opening of a new aquarium and notice a visitor slip and fall on the wet floor. Though he has fractured his neck, he is still alive and can be safely evacuated by paramedics as long as he is not moved. However, he has fallen on a tube supplying a separate underwater observation chamber with oxygen. There are five visitors currently in the observation chamber. Without oxygen supply, they will soon suffocate.<br><br>Pushing the injured man off the supply tube will kill him but the five visitors in the observation chamber will be supplied with oxygen again and survive.<br><br>Do you push the injured man off the supply tube in order to supply the observation chamber and its five visitors with oxygen?	You are at the opening of a new aquarium and notice a visitor slip and fall on the wet floor. Though he has fractured his neck, he is still alive and can be safely evacuated by paramedics as long as he is not moved. However, he has fallen on a supply tube used to regulate the temperature in a separate underwater observation chamber. There are five visitors currently in the observation chamber. If the heating system fails, the temperature within the observation chamber will drop drastically, causing the visitors to catch a cold.<br><br>Pushing the injured man off the supply tube will kill him but the five visitors in the observation chamber will not become sick.<br><br>Do you push the injured man off the supply tube in order to be able to regulate the temperature in the observation chamber currently hosting five visitors again?
**CU02**	You are at the opening of a new aquarium and notice a visitor slip and fall on the wet floor. Though he has fractured his neck, he is still alive and can be safely evacuated by paramedics as long as he is not moved. However, he has fallen on a tube supplying a separate underwater observation chamber with oxygen. There are five visitors currently in the observation chamber. Without oxygen supply, they will soon suffocate.<br><br>Pulling the supply tube out from under the injured man will kill him but the five visitors in the observation chamber will be supplied with oxygen again and survive.<br><br>Do you pull the supply tube out from under the injured man in order to supply the observation chamber and its five visitors with oxygen?	You are at the opening of a new aquarium and notice a visitor slip and fall on the wet floor. Though he has fractured his neck, he is still alive and can be safely evacuated by paramedics as long as he is not moved. However, he has fallen on a supply tube used to regulate the temperature in a separate underwater observation chamber. There are five visitors currently in the observation chamber. If the heating system fails, the temperature within the observation chamber will drop drastically, causing the visitors to catch a cold.<br><br>Pulling the supply tube out from under the injured man will kill him but the five visitors in the observation chamber will not become sick.<br><br>Do you pull the supply tube out from under the injured man in order to be able to regulate the temperature in the observation chamber currently hosting five visitors again?
**CU03**	You are standing on a pedestrian bridge over railway tracks and notice an empty, out-of-control freight car speeding towards five track workers that are doing repair work on the track. They cannot see the fast-approaching waggon and, due to the level of noise, cannot hear it either. Your foot is stuck in the railing but you can push the person standing next to you off the bridge and onto the rails. The freight car will run the person over and will be slowed down significantly, preventing it from running over the five track workers.<br><br>If you push the person, they will fall and be run over and killed by the freight car. This will slow down the waggon and save the five track workers.	You are standing on a pedestrian bridge over railway tracks and notice an empty, out-of-control freight car speeding towards a parked train. Five track workers are in the process of doing repair work on the parked train. They cannot see the fast-approaching waggon and, due to the level of noise, cannot hear it either. Your foot is stuck in the railing but you can push the person standing next to you off the bridge and onto the rails. The freight car will run the person over and will be slowed down significantly, preventing it from colliding with the parked train and severely injuring the five track workers.<br><br>If you push the person, they will fall and be run over and killed by the freight car. However, doing this will slow down the waggon and prevent the five workers from getting injured.

	Do you push the person next to you onto the tracks, causing them to be run over, in order to slow down the freight car and save the five track workers from being run over?	Do you push the person next to you onto the tracks, causing them to be run over, in order to slow down the freight car and prevent the workers from getting injured?
**CU04**	You are standing on a pedestrian bridge over railway tracks and notice an empty, out-of-control freight car speeding towards five track workers that are doing repair work on the track. They cannot see the fast-approaching waggon and, due to the level of noise, cannot hear it either. There is a lever next to you that lowers the pedestrian bridge. Operating this lever will cause a person leaning against the railing of the bridge to fall onto the tracks. The freight car will run this person over and will be slowed down significantly, preventing it from running over the five track workers.<br>If you operate the lever the person will fall onto the tracks and be run over and killed by the freight car. This will slow down the waggon and save the five track workers.<br>Do you operate the lever in order to lower the railing of the pedestrian bridge, causing the person to fall off the bridge and onto the tracks? This will slow down the freight car and save the five track workers from being run over?	You are standing on a pedestrian bridge over railway tracks and notice an empty, out-of-control freight car speeding towards a parked train. Five track workers are in the process of doing repair work on the parked train. They cannot see the fast-approaching waggon and, due to the level of noise, cannot hear it either. There is a lever next to you that lowers the pedestrian bridge. Operating this lever will cause a person leaning against the railing of the bridge to fall onto the tracks. The freight car will run the person over and will be slowed down significantly. This will prevent it from colliding with the parked train and severely injuring the five track workers.<br>If you operate the lever the person will fall onto the tracks and be run over and killed by the freight car. This will slow down the waggon and prevent the five workers from getting injured.<br>Do you operate the lever in order to lower the railing of the pedestrian bridge, causing the person to fall off the bridge and onto the tracks? This will slow down the freight car and prevent the workers from getting injured.
**CU05**	You are a fireman trying to rescue five children from a burning house. Only one window can be used to safely evacuate the children. However, it is locked and jammed. You have to smash this large, heavy window immediately. Otherwise, the five children will die. A man who has already been rescued is waiting for evacuation on the outer ledge of the window. You cannot help him at the moment.<br>You can smash the window. This will cause the man to fall off the window ledge. The man will most likely die but the five children can be saved.<br>Do you smash the window, thereby pushing the man off the ledge, in order to be able to save the five children?	You are a fireman trying to rescue the pets of an already rescued man from a burning house. Only one window can be used to safely evacuate the animals, four cats and two dogs. However, it is locked and jammed. You have to smash this large, heavy window immediately. Otherwise, the six pets will die. A man who has already been rescued is waiting for evacuation on the outer ledge of the window. You cannot help him at the moment.<br>You can smash the window. This will cause the man to fall off the window ledge. The man will most likely die but the six pets can be saved.<br>Do you smash the window, thereby pushing the man off the ledge, in order to be able to save the six pets?
**CU06**	You are a fireman trying to rescue five children from a burning house. Only one window can be used to safely evacuate the children. However, it is locked and jammed. You have to smash this large, heavy window immediately. Otherwise, the five children will die. A man who has already been rescued is waiting for evacuation on the outer ledge of the window. You cannot help him at the moment.	You are a fireman trying to rescue the pets of an already rescued man from a burning house. Only one window can be used to safely evacuate the animals, four cats and two dogs. However, it is locked and jammed. You have to smash this large, heavy window immediately. Otherwise, the six pets will die. A man who has already been rescued is waiting for evacuation on the outer ledge of the window. You cannot help him at the moment.

	You can smash the window. This will cause large shards of glass to fall. The falling shards of glass will most likely kill the man waiting for evacuation but you will be able to save the five children.<br><br>Do you smash the window, causing large shards of glass to fall on the man, in order to be able to save the children?	You can smash the window. This will cause large shards of glass to fall. The falling shards of glass will most likely kill the man waiting for evacuation but you will be able to save the six pets.<br><br>Do you smash the window, causing large shards of glass to fall on the man, in order to be able to save the six pets?
**CU07**	You are a fireman trying to rescue five children from a burning house. Only one window can be used to safely evacuate the children. However, it is locked and jammed. You have to break this large, heavy window immediately using a sufficiently large object. Otherwise, the five children will die. The only available object is a man that is in the process of exiting the house by climbing over a narrow wall leading to the neighboring house.<br><br>You can shove the man, causing him to fall off the wall and through the window below, shattering it. This will probably kill him but you will be able to save the five children.<br><br>Do you shove the man, causing him to fall off the wall and shatter the window, in order to be able to save the five children?	You are a fireman trying to rescue the pets of an already rescued man from a burning house. Only one window can be used to safely evacuate the animals, four cats and two dogs. However, it is locked and jammed. You have to smash this large, heavy window immediately using a sufficiently large object. Otherwise, the six pets will die. The only available object is a man that is in the process of exiting the house by climbing over a narrow wall leading to the neighboring house.<br><br>You can shove the man, causing him to fall off the wall and through the window below, shattering it. This will probably kill him but you will be able to save the six pets.<br><br>Do you shove the man, causing him to fall off the wall and shatter the window, in order to be able to save the six pets?
**CU08**	You are a fireman trying to rescue five children from a burning house. Only one window can be used to safely evacuate the children. However, it is locked and jammed. You have to break this large, heavy window immediately using a sufficiently large object. Otherwise, the five children will die. The only available object is a man that is in the process of exiting the house by climbing over a narrow wall leading to the neighboring house.<br><br>You can throw a piece of burning debris at the man. He will try to dodge the debris, lose his balance and most likely fall through the window below, shattering it.<br><br>Do you throw a piece of burning debris at the man, causing him to fall off the wall and shatter the window, in order to be able to save the five children?	You are a fireman trying to rescue the pets of an already rescued man from a burning house. Only one window can be used to safely evacuate the animals, four cats and two dogs. However, it is locked and jammed. You have to smash this large, heavy window immediately using a sufficiently large object. Otherwise, the six pets will die. The only available object is a man that is in the process of exiting the house by climbing over a narrow wall leading to the neighboring house.<br><br>You can throw a piece of burning debris at the man. He will try to dodge the debris, lose his balance and most likely fall through the window below, shattering it. This will most likely kill him but you will be able to save the six pets.<br><br>Do you throw a piece of burning debris at the man, causing him to fall off the wall and shatter the window, in order to be able to save the six pets?
**CU09**	You're working on the top floor of a construction site. You notice that the gearbox of a lifting platform at the end of a steel beam is about to break off. The lifting platform is fully extended. There are five workers on the platform that will fall and die if the gearbox breaks off. You can save the five workers by quickly running over the steel beam towards the gearbox to activate its emergency mechanism. However, another worker is standing between you and the gearbox.	You're working on the top floor of a construction site. You notice that the gearbox of a lifting platform at the end of a steel beam is about to break off. The lifting platform is fully extended. There is a worker on the platform that will fall and die if the gearbox breaks off. You can save the worker by quickly running over the steel beam towards the gearbox to activate its emergency mechanism. However, three other workers are standing between you and the gearbox. You

	You know that you will cause him to fall if you quickly run across the narrow steel beam. The noise level at the construction site is too high to warn the workers.<br><br>If you run across the steel beam to activate the emergency mechanism you will be able to save the five workers on the lifting platform but you will cause the other worker on the steel beam to fall and die.<br><br>Do you run across the narrow steel beam in order to prevent the workers on the lifting platform to fall?	know that you will cause them to fall if you quickly run across the narrow steel beam. The noise level at the construction site is too high to warn the workers.<br><br>If you run across the steel beam to activate the emergency mechanism you will be able to save the worker on the lifting platform but you will cause the other three workers on the steel beam to fall and die.<br><br>Do you run across the narrow steel beam in order to activate the emergency mechanism? This will cause the three workers to fall but it will prevent the worker on the lifting platform to fall.
**CU10**	You’re working on the top floor of a construction site. You notice that the gearbox of a lifting platform at the end of a steel beam is about to break off. The lifting platform is fully extended. There are five workers on the platform that will fall and die if the gearbox breaks off. You can save the five workers by quickly running over the steel beam towards the gearbox to activate its emergency mechanism. However, there is a barrier with another worker standing next to it between you and the gearbox. You know that the barrier will crash into the worker, causing him to fall if you run across the narrow steel beam quickly. The noise level at the construction site is too high to warn the workers.<br><br>If you run across the steel beam to activate the emergency mechanism you will be able to save the five workers but you will move the barrier which will then shove the worker off the steel beam, causing him to fall to his death.<br><br>Do you run across the narrow steel beam in order to activate the emergency mechanism that can prevent the five workers from falling off the lifting platform? You will move the barrier that causes the worker to fall.	You’re working on the top floor of a construction site. You notice that the gearbox of a lifting platform at the end of a steel beam is about to break off. The lifting platform is fully extended. There is a worker on the platform that will fall and die if the gearbox breaks off. You can save the worker by quickly running over the steel beam towards the gearbox to activate its emergency mechanism. However, there is a barrier with three other workers standing next to it between you and the gearbox. You know that the barrier will crash into the workers, causing them to fall if you run across the narrow steel beam quickly. The noise level at the construction site is too high to warn the workers.<br><br>If you run across the steel beam to activate the emergency mechanism you will be able to save the worker on the lifting platform but you will move the barrier which will then shove the three workers off the steel beam, causing them to fall to their death.<br><br>Do you run across the narrow steel beam in order to activate the emergency mechanism that can prevent the worker from falling off the lifting platform? You will move the barrier that causes the three workers to fall.
**CU11**	You are travelling on a motorboat with multiple passangers when you notice five swimmers in the distance that appear to be drowning. If you don’t race towards them at maximum speed you will not be able to reach them in time and all five of them will drown. To reach top speed, however, you must reduce the weight of the boat. The only possibility of doing this is by pushing a passenger that is standing by the edge of the flat deck overboard into the water. You know that this passenger cannot swim and will drown.<br><br>If you push the passenger overboard, you will cause him to drown but you will be able to save the five swimmers.	You are travelling on a motorboat with multiple passangers when you notice a swimmer in the distance that appears to be drowning. If you don’t race towards him at maximum speed you will not be able to reach him in time and he will drown. To reach top speed, however, you must reduce the weight of the boat. The only possibility of doing this is by pushing two passengers that are standing by the edge of the flat deck overboard into the water. You know that these passengers cannot swim and will drown.<br><br>If you push the passengers overboard, you will cause them to drown but you will be able to save the swimmer.

	Do you push the passenger overboard in order to achieve top speed and reach the swimmers in time?	Do you push the two passengers overboard in order to achieve top speed and reach the swimmer in time?
**CU12**	You are travelling on a motorboat with multiple passangers when you notice five swimmers in the distance that appear to be drowning. If you don’t race towards them at maximum speed you will not be able to reach them in time and all five of them will drown. The only possibility of doing this is by abruptly accelerating the boat. This will cause a passenger that is standing by the edge of the flat deck to fall overboard and into the water. You know that this passenger cannot swim and will drown.<br>If you accelerate the boat abruptly, you will cause the death of the passenger but you will also be able to save the five swimmers.<br>Do you abruptly accelerate the boat, causing the passenger to fall into the water, in order to achieve top speed and reach the five swimmers in time?	You are travelling on a motorboat with multiple passangers when you notice a swimmer in the distance that appears to be drowning. If you don’t race towards him at maximum speed you will not be able to reach him in time and he will drown. The only possibility of doing this is by abruptly accelerating the boat. This will cause two passengers that are standing by the edge of the flat deck to fall overboard and into the water. You know that these passengers cannot swim and will drown.<br>If you accelerate the boat abruptly, you will cause the death of the passengers but you will also be able to save the swimmer.<br>Do you abruptly accelerate the boat, causing the two passengers to fall into the water, in order to achieve top speed and reach the swimmer in time?
**CO01**	You are driving down a busy road when a young mother carrying a baby suddenly trips and threatens to fall onto the street. You are driving too fast to brake in time. Your only chance is to swerve to the side. However, you would have to swerve right into the path of an old lady walking by the side of the road.<br>If you swerve to the side abruptly to avoid hitting the young mother and her baby, you will collide with the old lady. This will kill her but save the young mother and her baby.<br>Do you abruptly swerve to the side to avoid hitting the young mother and her baby and steer the car into the old lady?	You are driving down a busy road when a young mother carrying a baby suddenly trips and threatens to fall onto the street. You are driving too fast to brake in time. Your only chance is to swerve to the side. However, you would have to swerve right into the path of a group of five schoolchildren.<br>If you swerve to the side abruptly to avoid hitting the young mother and her baby, you will collide with the five schoolchildren. This will kill them but save the young mother and her baby.<br>Do you abruptly swerve to the side to avoid hitting the young mother and her baby and steer the car into the five schoolchildren?
**CO02**	You are driving down a busy road when a young mother carrying a baby suddenly trips and threatens to fall onto the street. You are driving too fast to brake in time. Your only chance is to swerve to the side, right into a scaffolding. If you hit the scaffolding, it will collapse on top of an old lady that is walking by underneath and she will be killed.<br>If you swerve to the side abruptly to avoid hitting the young mother and her baby, you will hit the scaffolding. The scaffolding will collapse and kill the old lady but it will save the young mother and her baby.<br>Do you swerve to the side to avoid hitting the young mother and her baby and instead drive into the scaffolding, causing it to collapse?	You are driving down a busy road when a young mother carrying a baby suddenly trips and threatens to fall onto the street. You are driving too fast to brake in time. Your only chance is to swerve to the side, right into a scaffolding. If you hit the scaffolding, it will collapse on top of a group of five schoolchildren that are walking by underneath and they will be killed.<br>If you swerve to the side abruptly to avoid hitting the young mother and her baby, you will hit the scaffolding. The scaffolding will collapse and kill the five schoolchildren but it will save the young mother and her baby.<br>Do you swerve to the side to avoid hitting the young mother and her baby and instead drive into the scaffolding, causing it to collapse?
