# Supplementary material for: Effects of Instrumentality and Personal Force on Deontological and Utilitarian Inclinations in Harm-Related Moral Dilemmas
Source: Front Psychol. 2020 Jun 19;11:1222. doi: 10.3389/fpsyg.2020.01222 (PMC7318801; doi:10.3389/fpsyg.2020.01222)
Supplement: Supplementary file 2 [file Data_Sheet_2.PDF]

Online Supplementary Material: Power Analyses

Ludwig, J., Reisenzein, R., & Hiemisch, A. *Effects of instrumentality and personal force on deontological and utilitarian inclinations in harm-related moral dilemmas.*

Experiment 1

A sensitivity power analysis was conducted for a 2 (within, parameter type: PD Deontology vs. PD Utilitarianism) x 2 (between, instrumentality: means vs. side-effect) repeated measures ANOVA using *G*Power* (Faul, Erdfelder, Lang, & Buchner, 2007). It indicated a minimum detectable effect size of η_p^2 = .088, assuming α = .05, 1 – β = .80, and a correlation of *r* = -.45 between the repeated measures (i.e., the PD parameters), corresponding to the actually obtained correlation in this study. The detectable effect size corresponds to the typical effects of the experimental manipulations used in previous moral judgment PD studies (e.g., Conway & Gawronski, 2013, Study 2, reported an effect size of η_p^2 = .098 for the manipulation of cognitive load).

Experiment 2

A sensitivity power analysis of the planned 2 (within, parameter type: PD Deontology vs. PD Utilitarianism) x 2 (between, instrumentality: means vs. side-effect) x 2 (between, personal force: personal vs. impersonal) repeated measures ANOVA (G*Power; Faul et al., 2007) indicated a minimum detectable effect size of η_p^2 = .077, assuming α = .05, 1 – β = .80, and a correlation of *r* = -.29 among the repeated measures (as actually found in the experiment). Hence, the sample size assured appropriate levels of statistical power for detecting effects of the size found in Experiment 1 and in comparable prior work (e.g., Conway & Gawronski, 2013).

Experiment 3

Based on an a priori power analysis, we decided on a target sample size of *N* = 300 participants before data collection (see the pre-registration document). The power analysis was conducted using *G*Power* (Faul et al., 2007) for the planned 2 (within, parameter type: PD Deontology vs. PD Utilitarianism) x 2 (between, instrumentality: means vs. side-effect) x 2 (between, personal force: personal vs. impersonal) repeated measures ANOVA. For *N* = 300, α = .05, 1 – β = .80, and an assumed correlation between the dependent measures (the PD parameters) corresponding to that obtained in Experiment 2 (*r* = -.29), the minimum detectable effect size was found to be η_p^2 = .023, which is much smaller than the effects obtained in Experiments 1 and 2 and in previous comparable studies (e.g., Conway & Gawronski, 2013). Conversely, experimental effects equal to those found in Experiment 2 (*N* = 88, e.g., the instrumentality effect, η_p^2 = .173) would be detected with a power of 1.

References

Conway, P., & Gawronski, B. (2013). Deontological and utilitarian inclinations in moral decision making: A process dissociation approach. *Journal of Personality and Social Psychology*, *104*(2), 216–235. https://doi.org/10.1037/a0031021

Faul, F., Erdfelder, E., Lang, A. G., & Buchner, A. (2007). G*Power 3: A flexible statistical power analysis program for the social, behavioral, and biomedical sciences. *Behavior Research Methods*, *39*(2), 175–191. https://doi.org/10.3758/bf03193146
